# Surface Crystal Modification of Na_3_V_2_(PO_4_)_3_ to Cast Intermediate Na_2_V_2_(PO_4_)_3_ Phase toward High‐Rate Sodium Storage

**DOI:** 10.1002/advs.202306168

**Published:** 2023-11-23

**Authors:** Hui Zhang, Lei Wang, Linlin Ma, Yahui Liu, Baoxiu Hou, Ningzhao Shang, Shuaihua Zhang, Jianjun Song, Shuangqiang Chen, Xiaoxian Zhao

**Affiliations:** ^1^ Department of Chemistry, College of Science Hebei Agricultural University Baoding 071001 China; ^2^ Department of Chemical Engineering School of Environmental and Chemical Engineering Shanghai University Shanghai 200444 P. R. China; ^3^ National Engineering Research Center of green recycling for strategic metal resources Institute of Process Engineering Chinese Academy of Sciences Beijing 100190 P. R. China; ^4^ College of Physics Qingdao University Qingdao 266071 P. R. China

**Keywords:** cathode, Na_3_V_2_(PO_4_)_3_, NASICON, sodium ion batteries, surface modification

## Abstract

The two‐phase reaction of Na_3_V_2_(PO_4_)_3_ – Na_1_V_2_(PO_4_)_3_ in Na_3_V_2_(PO_4_)_3_ (NVP) is hindered by low electronic and ionic conductivity. To address this problem, a surface‐N‐doped NVP encapsulating by N‐doped carbon nanocage (N‐NVP/N‐CN) is rationally constructed, wherein the nitrogen is doped in both the surface crystal structure of NVP and carbon layer. The surface crystal modification decreases the energy barrier of Na^+^ diffusion from bulk to electrolyte, enhances intrinsic electronic conductivity, and releases lattice stress. Meanwhile, the porous architecture provides more active sites for redox reactions and shortens the diffusion path of ion. Furthermore, the new interphase of Na_2_V_2_(PO_4_)_3_ is detected by in situ XRD and clarified by density functional theory (DFT) calculation with a lower energy barrier during the fast reversible electrochemical three‐phase reaction of Na_3_V_2_(PO_4_)_3_ – Na_2_V_2_(PO_4_)_3_ – Na_1_V_2_(PO_4_)_3_. Therefore, as cathode of sodium‐ion battery, the N‐NVP/N‐CN exhibited specific capacities of 119.7 and 75.3 mAh g^−1^ at 1 C and even 200 C. Amazingly, high capacities of 89.0, 86.2, and 84.6 mAh g^−1^ are achieved after overlong 10000 cycles at 20, 40, and 50 C, respectively. This approach provides a new idea for surface crystal modification to cast intermediate Na_2_V_2_(PO_4_)_3_ phase for achieving excellent cycling stability and rate capability.

## Graphical abstract

A surface‐N‐doped Na_3_V_2_(PO_4_)_3_ (NVP) encapsulating by N‐doped carbon nanocage (N‐NVP/N‐CN) is constructed, which decreases the energy barrier of Na^+^ diffusion from bulk to electrolyte, enhances intrinsic electronic conductivity, and releases the lattice stress. Furthermore, this structure enables a fast reversible electrochemical three‐phase reaction of Na_3_V_2_(PO_4_)_3_ – Na_2_V_2_(PO_4_)_3_ – Na_1_V_2_(PO_4_)_3_ for excellent cycling stability and rate capability as cathode of sodium‐ion battery.

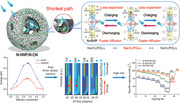

## 1 Introduction

Recently, sodium‐ion battery (SIB), as a promising alternative energy storage system to lithium‐ion battery (LIB), has attracted much attention due to its lower price, abundant resources, and similar electrochemical reaction mechanism to LIB.^[^ ^1^ ^]^ However, compared to lithium‐ion (*r* = 0.69 Å), sodium ion with a larger radius (*r* = 0.98 Å) leads to large volume variations and lattice stress, which greatly limits the development of SIB.^[^ ^2^ ^]^ Many cathode materials, including layered sodium transition metal oxides, Prussian blue and its analogs, polyanionic compounds, have been developed for SIB, but each of them has its own problem,^[^ ^3^ ^]^ and further research on cathode is needed. Na_3_V_2_(PO_4_)_3_ (NVP) with a theoretical capacity of 117.6 mAh g^−1^ (energy density of 401 Wh kg^−1^) is composed by the [VO_6_] regular octahedron and [PO_4_] tetrahedron, connected by oxygen at the vertex to form a “lantern‐shaped” structural unit of [V_2_(PO_4_)_3_],^[^ ^4^ ^]^ providing an ordered 3D open framework with a sodium superionic conductor (NASICON). Two Na^+^ cations can intercalate/deintercalate in each NVP cell at the working voltage of 3.3–3.4 V reversibly.^[^ ^5^ ^]^ Therefore, NVP is regarded as a promising cathode material for SIB with superior thermal stability.^[^ ^6^ ^]^ Whereas, its sluggish electron migration rate and the slow widely believed two‐phase reaction of Na_3_V_2_(PO_4_)_3_ – Na_1_V_2_(PO_4_)_3_ system in NVP bring insufficient capacity at high current density, limiting the further development in energy and power density of devices.^[^ ^7^, ^8^ ^]^

Improving intrinsic electronic conductivity and Na^+^ diffusion kinetic of NVP can facilitate the normally regarded two‐phase electrochemical reaction of Na_3_V_2_(PO_4_)_3_ – Na_1_V_2_(PO_4_)_3_ in Na_3_V_2_(PO_4_)_3_, which is an effective strategy to promote rate capability and cycling stability.^[^ ^9^ ^]^ Many researchers put their attention on the improvement of electronic conductivity and Na^+^ diffusion for NVP.^[^ ^10^ ^]^ Surface modification of lattice structure could promote Na^+^ diffusion from the bulk to electrolyte and confine the structural collapse during the phase transformation from NVP to NaV_2_(PO_4_)_3_.^[^ ^11^, ^12^ ^]^ Besides, surface modification such as doping N atom in the crystal lattice on the surface is applied to improve intrinsic electronic conductivity via adjusting band structure, which benefits rate capability.^[^ ^13^, ^14^ ^]^

Another efficient approach to improve electronic conductivity and Na^+^ diffusion is embedding active material into a carbon matrix to construct a porous and hollow structure.^[^ ^15^, ^16^ ^]^ For one thing, the abundant pores in materials can provide sequential channels for the permeation of electrolytes and increase the contacting area with electrolytes,^[^ ^17^, ^18^ ^]^ which largely shortens the Na^+^ diffusion path and promotes more active sites for the redox reaction.^[^ ^19^, ^20^, ^21^ ^]^ The porous structure can promote Na^+^ diffusion kinetic in NVP. Furthermore, the introduction of carbon material can markedly promote electron transportation between NVP particles.^[^ ^22^ ^]^ The fast electron transfer rate and Na^+^ diffusion kinetic facilitate the reversible electrochemical reaction of NVP for excellent rate capability.^[^ ^23^, ^24^ ^]^

Nonetheless, the improvement of Na^+^ diffusion kinetic in NVP runs into obstacles based on the widely believed two‐phase electrochemical reaction between Na_3_V_2_(PO_4_)_3_ and Na_1_V_2_(PO_4_)_3_. Masquelier et al. reported an apparent intermediate Na_2_V_2_(PO_4_)_3_ by operando synchrotron X‐ray diffraction, demonstrating a three‐phase reaction of Na_3_V_2_(PO_4_)_3_ – Na_2_V_2_(PO_4_)_3_ – Na_1_V_2_(PO_4_)_3_ system. Different from the two‐phase reaction, in the three‐phase reaction of Na_3_V_2_(PO_4_)_3_ – Na_2_V_2_(PO_4_)_3_ – Na_1_V_2_(PO_4_)_3_ system, the lattice mismatch between Na_3_V_2_(PO_4_)_3_ and Na_1_V_2_(PO_4_)_3_ was reduced to promote fast phase transition.^[^ ^25^ ^]^ However, there is currently no in‐depth research on the three‐phase reactions of NVP. It is urgent to find a delicate way to precisely control the nanostructure to realize the fast reversible three‐phase reaction of Na_3_V_2_(PO_4_)_3_ – Na_2_V_2_(PO_4_)_3_ – Na_1_V_2_(PO_4_)_3_ system.

**Figure 1:**
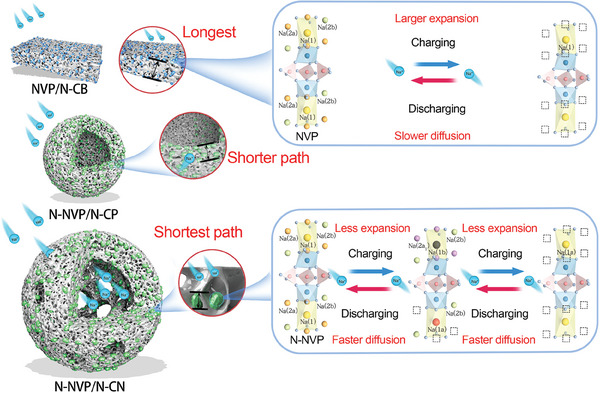
Schematic illustration of the advantages of nanocage structure and surface crystal modification of N‐NVP/N‐CN on electron and Na^+^ diffusion rate for fast reversible three‐phase reaction.

Herein, we rationally constructed surface‐N‐doped NVP nanoparticles encapsulated in the porous N‐doped carbon nanocage (N‐NVP/N‐CN) to enhance the intrinsic electronic conductivity and Na^+^ diffusion kinetics by a scalable spray drying method. During the synthesis process, nitrogen is randomly doped in both the surface crystal structure of NVP and the carbon layer. The surface crystal modification of NVP decreases the energy barrier of Na^+^ diffusion from bulk to electrolyte, enhances intrinsic electronic conductivity, and releases the lattice stress to increase mechanical stability during circulation. Besides, as shown in **Figure** **1**, the porous and hollow structure increases the contacting area between the material and the electrolyte, providing more active sites for redox reactions and shortening the ionic diffusion path. A new interphase of Na_2_V_2_(PO_4_)_3_ was clarified by in situ X‐ray diffraction patterns and confirmed a lower energy barrier with this interphase by the density functional theory (DFT) calculations during the redox reactions in N‐NVP/N‐CN due to its fast Na^+^ and electron diffusion. It illustrates the fast reversible electrochemical three‐phase reaction of Na_3_V_2_(PO_4_)_3_ – Na_2_V_2_(PO_4_)_3_ – Na_1_V_2_(PO_4_)_3_ system, while the phase transition of pure NVP is showing an apparent hysteresis effect. Consequently, as the cathode material of SIB, the N‐NVP/N‐CN displays a higher specific capacity, excellent rate capability, and cycling stability.

## 2 Results and Discussion

**Figure 2:**
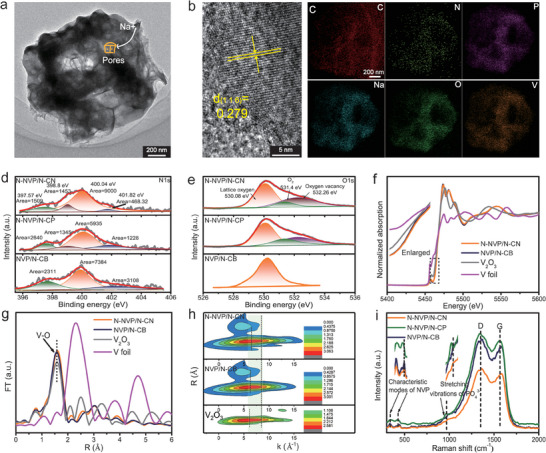
a) The TEM image, b) HRTEM, and c) element mapping distribution of N‐NVP/N‐CN; d, e) The XPS high‐resolution analysis of N1s and O1s, f) the K edge XANES spectra of V, g) R space Fourier‐transforms of EXAFS, h) wavelet transforms and i) Raman spectrum of N‐NVP/N‐CN, N‐NVP/N‐CP, and NVP/N‐CB.

The N‐NVP/N‐CN was fabricated by a simple spray drying and calcination process as illustrated in Figure S1 (Supporting Information), and the N‐doped NVP/N‐C hollow polyhedron (N‐NVP/N‐CP) and NVP/N‐C bulk (NVP/N‐CB) were taken as a comparison. As shown in **Figure** **2**a, and Figures S2 and S3a (Supporting Information), the precursor of N‐NVP/N‐CP own a smooth surface with potholes, which was retained after calcination. However, the precursor of N‐NVP/N‐CN exhibited a shrink structure outside with the polystyrene (PS) spheres sealed inside of the shell, the hollow nanocage with a porous shrinking shell was formed due to the decomposition of PS spheres after calcination. This unique nanocage structure could provide short channels for Na^+^ diffusion and the permeation of electrolyte, offering more active sites for redox reactions.^[^ ^26^, ^27^ ^]^ Meanwhile, the porous carbon shell originating from carbonization of polyvinylpyrrolidone (PVP) was observed clearly (Figures S3b and S4, Supporting Information). Enlarging the boundary of NVP particle and carbon shell, the interplanar spacing of 0.279 nm attributed to (116) crystal plane of NVP^[^ ^28^, ^29^ ^]^ was detected clearly in Figure 2b and Figure S3c (Supporting Information). The carbon content was evaluated by the thermogravimetric analysis (TGA) in air gas. As shown in Figure S5 (Supporting Information), the carbon content of NVP/N‐CB, N‐NVP/N‐CP, or N‐NVP/N‐CN was calculated to be 12%, 12.9%, or 6.5%, respectively. The tap density of NVP/N‐CB, N‐NVP/N‐CP, and N‐NVP/N‐CN were calculated to be 1.34, 1.18, and 1.26 g cm^−3^ as shown in Figure S6 (Supporting Information). Besides, the nitrogen adsorption/desorption isotherms of N‐NVP/N‐CN, N‐NVP/N‐CP, and NVP/N‐CB were carried out in Figure S7 (Supporting Information), which displays the specific surface areas are 39.27, 31.34, and 57.68 m^2^ g^−1^, respectively. Additionally, the element mapping by transmission electron microscopy (TEM) was applied to analyze the element distribution of N‐NVP/N‐CN and N‐NVP/N‐CP (Figure 2c; Figures S8 and S3d, Supporting Information). Combining with the results of energy spectrum analysis (Figure S9, Supporting Information), six elements of Na, V, P, O, C, and N were detected. Four elements of Na, V, P, and O were distributed in NVP particles uniformly, which were wrapped by the C element. Interestingly, the N element was found in both NVP particles and the carbon layer.^[^ ^30^ ^]^

To verify whether the nitrogen was doped into the lattice of NVP or carbon layer only, many spectral characterizations were performed. The X‐ray photon spectroscopy (XPS) was used to analyze the electronic structure information of elements. Six elements of P, C, N, V, O, and Na were observed in the full pattern of XPS in Figures S10 and S11 (Supporting Information). Three types of N1s, including pyridine type (398.04 eV), pyrrole type (400.02 eV), and graphite type (402.5 eV),^[^ ^31^ ^]^ were detected in high‐resolution curves of N1s (Figure 2d). According to the relative integral area of N1s as shown in Table S1 (Supporting Information), the N‐NVP/N‐CN achieved a higher ratio of pyridine type to graphite type, which was good for conductivity.^[^ ^32^ ^]^ Especially, a metal type N in N‐NVP/N‐CP and N‐NVP/N‐CN was detected, confirming the introduction of N atom to the lattice of NVP.^[^ ^33^ ^]^ Compared to NVP/N‐CB, the peaks corresponding to V2p1/2, V2p3/2 (Figure S12, Supporting Information)^[^ ^34^, ^35^ ^]^ in N‐NVP/N‐CN or N‐NVP/N‐CP were shifted to the high binding energy, illustrating partial O was replaced by N with a lower electronegativity (Figure 2e), resulting in an increased attraction of V with electron.^[^ ^36^ ^]^ The extended X‐ray absorption fine structure (EXAFS) analyses and X‐ray absorption near‐edge structure (XANES) were further performed to characterize the electronic structure information of V. Figure 2f depicted V K‐edge XANES spectra of N‐NVP/N‐CN, NVP/N‐CB, V_2_O_3_, and V foil using V^0^ as the standard oxidation state. Compared to NVP/N‐CB and V_2_O_3_, a higher pre‐edge of N‐NVP/N‐CN has signified the higher valence of V due to the introduction of N.^[^ ^37^ ^]^ Furthermore, the peak position at 1.60 Å in N‐NVP/N‐CN by the Fourier‐transforms of EXAFS analyses (Figure 2g) was higher than that of NVP/N‐CB and V_2_O_3_, suggesting a longer V─O bond resulted from the N‐doping.^[^ ^38^ ^]^ The coordination environment was demonstrated by wavelet transform as shown in Figure 2h. The maximum coordination center of V in N‐NVP/N‐CN at 5.94 Å^−1^ was detected, proving the coordination environment of V─O was changed due to N‐doping.^[^ ^39^ ^]^ The X‐ray diffraction (XRD), fourier transform infrared spectroscopy (FTIR), and Raman spectrums were recorded to clarify the changes in lattice and functional groups. As shown in XRD (Figure S13, Supporting Information), the Na_3_V_2_(PO_4_)_3_ (JCPDS#53‐0018)^[^ ^40^ ^]^ was identified, wherein the peak position of (2 0−4) in N‐NVP/N‐CN and N‐NVP/N‐CP shifted to a high angle slightly compared to NVP/N‐CB, meaning the lattice contraction due to the replacement of the O on the surface of [PO_4_] tetrahedron or the [PO_4_] tetrahedron by N directly.^[^ ^11^ ^]^ It was testified by the XRD refinement as shown in Figure S14, and Tables S2–S4 (Supporting Information), and the highest N/O ratio of 2.92/13.61 in N‐NVP/N‐CN according to the XPS results (Table S5). Meanwhile, as shown in FTIR of Figure S15 (Supporting Information), the peaks at 567.4 and 1044.3 cm^−1^ attributed to vibration of P‐O and 1186.3 cm^−1^ attributed to PO_4_^3−^ unsymmetrical stretching vibration in N‐NVP/N‐CN were blueshifted compared to N‐NVP/N‐CP or NVP/N‐CB.^[^ ^41^ ^]^ In Raman spectrum (Figure 2i), the characteristic peak of [VO_6_] at 426 cm^−1^ and stretching vibration^[^ ^42^ ^]^ of PO_4_^3−^ at 990 cm^−1^ in N‐NVP/N‐CN were blueshifted compared to NVP/N‐CB. The above results both suggested that the [VO_6_] and PO_4_^3−^ units were shrunk due to doping N atom.^[^ ^43^ ^]^ Therefore, the N was successfully doped into NVP lattice of N‐NVP/N‐CN but the NVP/N‐CB, which promotes the diffusion kinetics of Na^+^,^[^ ^44^ ^]^ benefiting the rate capability and cycling stability.

**Figure 3:**
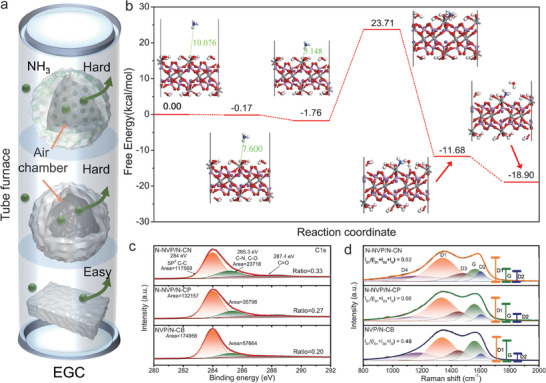
a) The model of the EGC influencing the N‐doping to NVP lattice; b) The curve of diffusion barriers at various transition states from Na_6_V_4_P_6_O_24_(H_2_O)_6_ to Na_6_V_4_P_6_O_24_(H_2_O)_5_NH_3_ through the reaction between O in vanadium oxygen octahedron on the surface of NVP with NH_3_; c) The XPS high‐resolution analysis curves of C1s; d) The peaks analysis of D‐band and G‐band in Raman spectrum by curve fitting of N‐NVP/N‐CN, N‐NVP/N‐CP, and NVP/N‐CB.

This interesting phenomenon that the N was doped into the lattice of NVP is aroused by the effect of gas chamber (EGC). NH_3_ was generated during the pyrolysis of ammonium salt and PVP as shown in **Figure** **3**a. For the NVP/N‐CB system, the generated NH_3_ was easy to escape without the enclosed carbon shell. In contrast, in N‐NVP/N‐CP or N‐NVP/N‐CN system, NH_3_ gas was trapped by the enclosed carbon hollow shell, forming a “nano gas chamber”, leading to a high concentration of NH_3_ gas in the chambers, which was confirmed by the slower mass losing in TGA test of N‐NVP/N‐CP or N‐NVP/N‐CN compared to NVP/N‐CB (Figure S16, Supporting Information). Thus, the increased kinetic energy of NH_3_ molecule was endowed to increase collision activation energy^[^ ^45^ ^]^ with NVP during calcination, which triggered the N‐doping in the lattice of NVP.

Besides, the specific position of N‐doping was clarified by the DFT calculation. Two approaches were performed and compared. First, various transition states of O in NVP replaced by N during the reaction with NH_3_ were calculated. As shown in Figure S17 (Supporting Information), an ultra‐high diffusion barrier of 172.1 kcal mol^−1^ was obtained, which proved the reaction of NH_3_ with O in NVP directly was impossible. Another approach was designed, wherein the lattice of NVP was sliced along with the (0 0 1) surface of the structure Na_3_V_2_(PO_4_)_3_ and the O atoms at the surface were saturated by H atom to form Na_6_V_4_P_6_O_24_(H_2_O)_6_ for balancing the charge. The diffusion barriers at various transition states from Na_6_V_4_P_6_O_24_(H_2_O)_6_ (NVP) to Na_6_V_4_P_6_O_24_(H_2_O)_5_NH_3_ (N‐NVP) through the reaction between NH_3_ and O in vanadium oxygen octahedron on the surface of NVP were calculated. As shown in Figure 3b, a lower diffusion barrier of 23.71 kcal mol^−1^ was achieved, proving O in vanadium oxygen octahedron on the surface of NVP lattice can be replaced by the N atom to form N‐NVP. Meanwhile, the EGC influenced the N‐doping content in the carbon layer as well, which was clarified by the XPS high‐resolution analysis. As shown in Figure 2d, a higher ratio of pyridine and pyrrole types in N‐NVP/N‐CN was achieved based on the relative integral area. Meanwhile, three characteristic peaks of C1s corresponding to 284.0, 285.3, and 287.4 eV were attributed to C─C, C─O, or C─N, and C═O bonds in Figure 3c.^[^ ^46^, ^47^ ^]^ The larger integral area of C─N in N‐NVP/N‐CP or N‐NVP/N‐CN has proved the abundant doped N atom in carbon shell, leading to faster electron transmission.^[^ ^48^ ^]^ Additionally, the structural characteristic of the carbon layer was explored by Raman spectrum. Two border peaks around 1350 and 1590 cm^−1^ were identified as D‐band and G‐band,^[^ ^49^ ^]^ which can be divided into five peaks as D1, D2, D3, D4, and G‐band by curve fitting. Wherein, the degree of disorder in the carbon layer was indicated by the intensity ratio of I_D1_/(I_G_ +I_D1_+I_D2_).^[^ ^50^ ^]^ As shown in Figure 3d, it was mentioned that the N‐NVP/N‐CN exhibited a higher value of I_D1_/(I_G_ +I_D1_+I_D2_) (0.52) compared to NVP/N‐CB (0.46), suggesting an efficient doping nitrogen into carbon layer.^[^ ^51^ ^]^ Thus, the high ratio of nitrogen was doped into the carbon layer, facilitating a rapid electron transfer.^[^ ^52^, ^53^ ^]^

**Figure 4:**
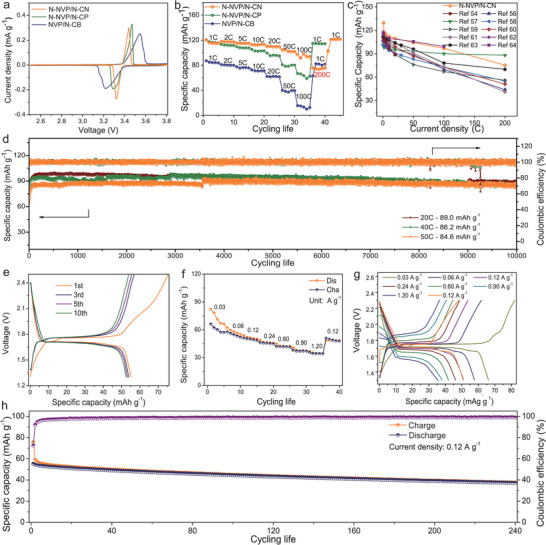
The electrochemical performance of N‐NVP/N‐CN, N‐NVP/N‐CP, and NVP/N‐CB as cathode in a half‐cell system. a) The CV curves at a scan rate of 0.1 mV s^−1^; b) The cycling stability at different current densities of 1, 2, 5, 10, 20, 50, 100, 200, and 1 C, respectively; c) The comparison of rate capability with other refs;^[^ ^54^, ^56^, ^57^, ^58^, ^59^, ^60^, ^61^, ^62^, ^63^, ^64^ ^]^ d) The long cycling stability at 20, 40, and 50 C of N‐NVP/N‐CN; The electrochemical performance of N‐NVP/N‐CN//N‐NVP/N‐CN symmetric full‐cell. e) The galvanostatic charging/discharging curves at a current density of 0.12 A g^−1^ in the 1st, 3rd, 5th, and 10th cycles; f) The rate performance at different current densities; g) the corresponding charging/discharging profiles; h) The long cycling performance at 0.12 A g^−1^.

The electrochemical properties of N‐NVP/N‐CN (with 600 mg PS spheres as shown in Figure S18, Supporting Information), N‐NVP/N‐CP, and NVP/N‐CB were measured by using a standard half‐cell configuration. First, the cyclic voltammograms (CV) with a voltage range of 2.5–3.8 V were applied to explore the electrochemical behavior. As shown in **Figure** **4**a, a pair of redox peaks at 3.32 V (cathodic peak) and 3.43 V (anodic peak) in N‐NVP/N‐CN with a smaller difference between cathodic and anodic peaks were detected. Correspondingly, a pair of charging and discharging platforms with smaller voltage differences at 1 C was shown in Figure S19a (Supporting Information), signifying a smaller polarization, as well as CV curves after 1st, 5th, 10th, and 30th cycles in Figure S20 (Supporting Information).^[^ ^54^ ^]^ Compared to N‐NVP/N‐CP and NVP/N‐CB, the N‐NVP/N‐CN exhibited higher specific capacities (119.7, 117.5, 115.1, 112.3, 109.6, 104.3, and 96.9 mAh g^−1^ at 1, 2, 5, 10, 20, 50, and 100 C, even up to 200 C with a capacity of 75.3 mAh g^−1^ in Figure 4b). When the current density was back to 1 C, a high specific capacity of 120.8 mAh g^−1^ was still obtained with an obvious charging and discharging platform (Figure S21, Supporting Information), proving an excellent rate capability. The contact area between electrode material and electrolyte was improved due to permeation of electrolyte, resulting in the higher specific capacity at 2 C or 5 C than 1 C.^[^ ^55^ ^]^ As shown in Figure 4c, compared to previously reported works,^[^ ^54^, ^56^, ^57^, ^58^, ^59^, ^60^, ^61^, ^62^, ^63^, ^64^ ^]^ the N‐NVP/N‐CN has shown large advantages at both low and high rates. Furthermore, N‐NVP/N‐CN exhibited excellent cycling stability with a high specific capacity of 119.7 mAh g^−1^ after 100 cycles in Figure S19b (Supporting Information). The specific capacity at 1 C, slightly higher than the theoretical specific capacity of NVP, is related to the porous structure of the material, which providing storage space for sodium ions corresponding capacitance contribution.^[^ ^65^ ^]^ Even when the current density was increased to 20, 40, 50, and 100 C, high capacities of 89.0, 86.2, 84.6, and 64.4 mAh g^−1^ were still maintained after 10 000 cycles in Figure 4d and Figure S22 (Supporting Information). At the first several cycles, the contact between electrode material and electrolyte was improved due to permeation of electrolyte, resulting in increase of specific capacity.^[^ ^66^ ^]^ Shockingly, the capacity can be maintained at 59.4 mAh g^−1^ even after 200 cycles at a high current density of 200 C (Figure S23, Supporting Information). To verify the practical application possibility, N‐NVP/N‐CN was measured at different environment temperatures (−10 to 50 °C) and different loading masses as shown in Figure S24 (Supporting Information). Obviously, the N‐NVP/N‐CN demonstrated better thermal adaptability when the loading mass was 1.75 mg cm^−2^. Finally, the electrochemical performance of the N‐NVP/N‐CN//N‐NVP/N‐CN symmetric device was checked. The symmetric device demonstrated a long and stable charging or discharging platform within a voltage range from 1.3 to 2.5 V depicted in Figure 4e and Figure S25 (Supporting Information), which was good for stable energy output. Meanwhile, the rate capability was measured by a galvanostatic charging/discharging test as shown in Figure 4f. The specific capacities of 66.1, 57.6, 51.0, 46.2, 41.9, and 37.9 mAh g^−1^ were obtained at current densities of 0.03, 0.06, 0.12, 0.24, 0.60, and 0.90 A g^−1^, and a reversible capacity of 35.6 mAh g^−1^ was still maintained at a high current density of 1.20 A g^−1^. When the current density was returned to 0.12 A g^−1^, a reversible capacity of 50.8 mAh g^−1^ was recovered. Furthermore, a long and stable charging or discharging platform at different current densities was acquired as shown in Figure 4g, even at the high current density of 1.20 A g^−1^, which illustrated its excellent rate capability. According with the rate test, the energy density and power density of the symmetric full cell was calculated, which exhibited superior comparing the Ragone plot with the previous reports (Figure S26, Supporting Information). The cycling stability was checked by a galvanostatic charging/discharging test at 0.12 A g^−1^. As demonstrated in Figure 4h, an initial specific capacity of 55.3 mAh g^−1^ was achieved, maintaining at 37.6 mAh g^−1^ after 240 cycles. Therefore, the N‐NVP/N‐CN exhibited a high specific capacity, excellent rate capability, and cycling stability in both the half cell system and N‐NVP/N‐CN//N‐NVP/N‐CN symmetric device.

**Figure 5:**
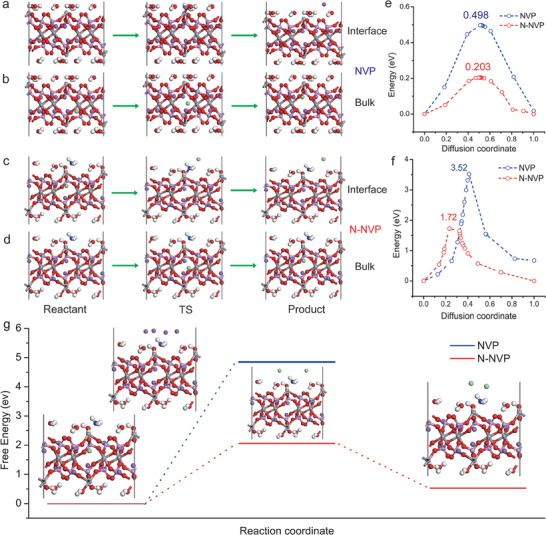
a) The DFT calculation model of Na^+^ diffusion from bulk to surface and b) in bulk of NVP; c) The DFT calculation model of Na^+^ diffusion from bulk to surface and d) in bulk of N‐NVP; e) The energy profiles of Na^+^ diffusion in bulk and f) from bulk to surface along the minimum energy path of NVP or N‐NVP; g) The diffusion barriers from Na_6_V_4_P_6_O_24_(H_2_O)_5_NH_3_ to Na_2_V_4_P_6_O_24_(H_2_O)_5_NH_3_ directly and from Na_6_V_4_P_6_O_24_(H_2_O)_5_NH_3_ to Na_4_V_4_P_6_O_24_(H_2_O)_5_NH_3_ then to Na_2_V_4_P_6_O_24_(H_2_O)_5_NH_3_.

The reason to brilliant electrochemical performance of N‐NVP/N‐CN was discussed in detail as follows. First, the shrinking surface, and vast pores on the surface offer multi‐channel for permeation of electrolyte, which provides more active sites for redox reaction and shorten diffusion path of electron and Na^+^ as shown in Figure 1. To prove the hypothesis, the capacitive contribution was analyzed by fitting CV curves at scan rates from 0.05 to 0.5 mV s^−1^ (Figure S27, Supporting Information). The N‐NVP/N‐CN exhibited a larger b value (0.5 ≤ b ≤ 1.0) as shown in Figure S28 (Supporting Information), signifying more capacitive contribution (Figures S29 and S30, Supporting Information) resulting from more electrochemical active area. Second, the surface modification of NVP lattice resulting from EGC improves Na^+^ and electron diffusion kinetic for the fast reversible three‐phase reaction of Na_3_V_2_(PO_4_)_3_ – Na_2_V_2_(PO_4_)_3_ – Na_1_V_2_(PO_4_)_3_. Taking NVP and N‐NVP as original models, the Na^+^ diffusion from bulk to the surface and in bulk was investigated by DFT calculation.^[^ ^67^ ^]^ The lattice innovations of NVP and N‐NVP from the reactant to the transition state and then to the product were described as shown in **Figure** **5**a–d. Compared with NVP (Figure 5e,f), the N‐NVP exhibited lower Na^+^ diffusion barrier energy of 1.72 eV from bulk to surface and 0.203 eV through bulk, which could provide solid evidence for faster Na^+^ diffusion kinetics of N‐NVP to foster excellent rate capability.^[^ ^68^ ^]^ Therefore, less interfacial impedance (*R*_ct_) identified by the semicircle in the high‐frequency region (Figure S31a, Supporting Information), and the larger Na^+^ diffusion coefficient of N‐NVP/N‐CN calculated from the slop of Warburg factor (Figure S31b, Supporting Information) or galvanostatic intermittent titration technique (GITT) as shown in Figure S32 (Supporting Information) were achieved. Furthermore, the surface crystal modification by doping nitrogen can adjust the band structure, which was evaluated by the total density of states (DOS). As shown in Figure S33 (Supporting Information), according to the density difference at the Fermi level,^[^ ^69^ ^]^ obviously the bandgap of N‐NVP of 1.914 eV is smaller than that of NVP (2.40 eV), largely enhancing the electron density at the Fermi level. Besides, the N‐doped carbon can change the electronic state of the carbon layer to promote the rapid transmission of the electron.^[^ ^70^, ^71^ ^]^ The fast Na^+^ diffusion kinetic and electronic conductivity of N‐NVP/N‐CN can facilitate the formation of intermediate Na_2_V_2_(PO_4_)_3_ to reduce the lattice mismatch between Na_3_V_2_(PO_4_)_3_ and Na_1_V_2_(PO_4_)_3_ for fast phase transition. Depicted in Figure 5g, the transformation free energy from N‐NVP to Na_2_V_4_P_6_O_24_(H_2_O)_5_NH_3_ (corresponding to from Na_3_V_2_(PO_4_)_3_ and Na_1_V_2_(PO_4_)_3_) was as high as 4.85 eV, the highest transformation free energy taking Na_4_V_4_P_6_O_24_(H_2_O)_5_NH_3_ as the intermediate state (corresponding to three‐phase reaction of Na_3_V_2_(PO_4_)_3_ – Na_2_V_2_(PO_4_)_3_ – Na_1_V_2_(PO_4_)_3_) was only 2.06 eV. It proved the fast reversible three‐phase reaction of Na_3_V_2_(PO_4_)_3_ – Na_2_V_2_(PO_4_)_3_ – Na_1_V_2_(PO_4_)_3_ was preferable in N‐NVP, which benefited rate capability and cycling stability.

**Figure 6:**
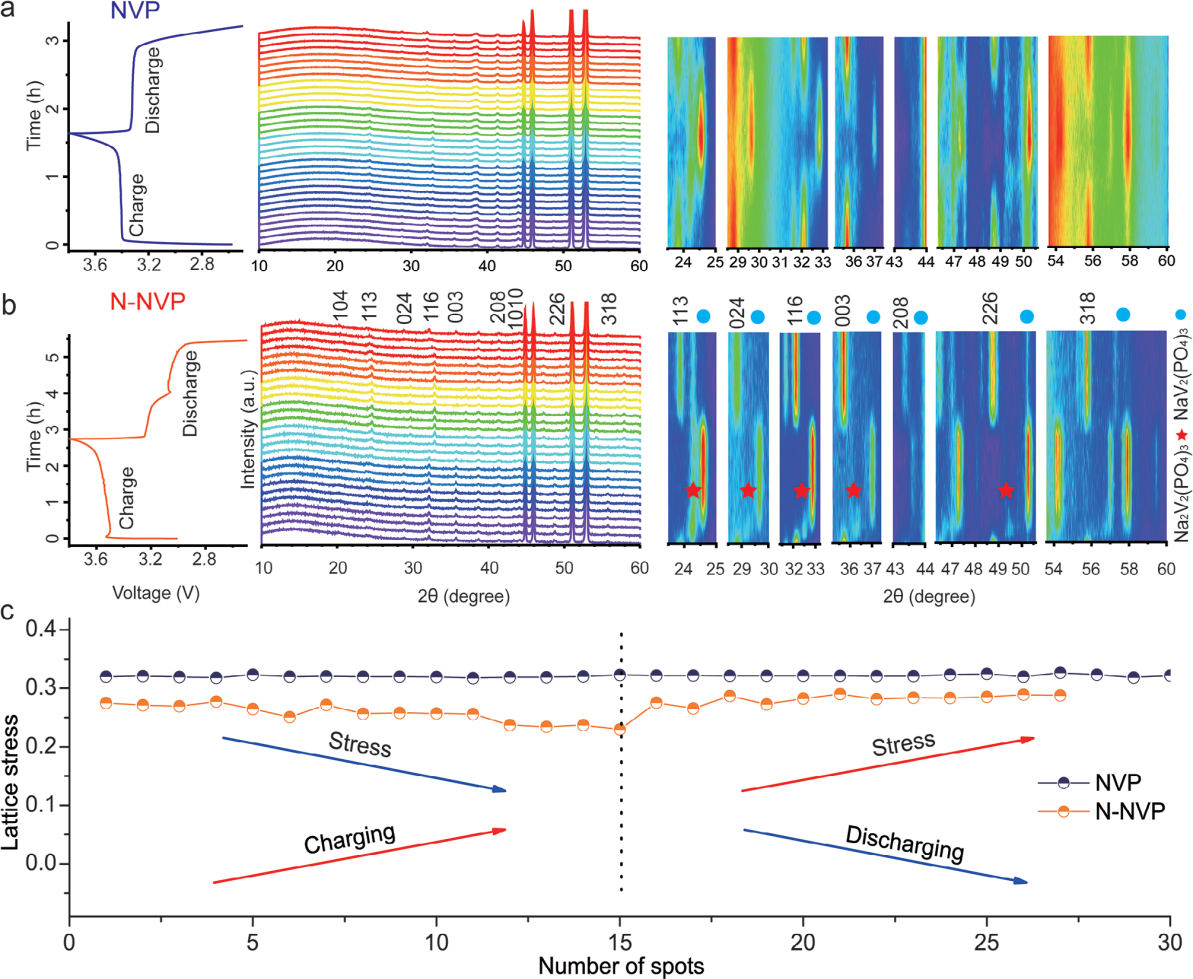
a) The in situ XRD curve of NVP/N‐CB and b) N‐NVP/N‐CN during the charging and discharging process; c) The calculation on lattice stress of NVP/N‐CB and N‐NVP/N‐CN according to in situ XRD.

To further analyze the advantage of reversible three‐phase reaction evolution and stress changes during the sodiation and desodiation processes, in situ XRD measurement was performed during a galvanostatic charge‐discharge process at 0.3 C between 2.5 and 4.0 V (vs Na^+^/Na) with simultaneous collections of XRD patterns. First, the structural evolutions of NVP/N‐CB (the main phase is NVP) and N‐NVP/N‐CN (the main phase is N‐NVP) are exhibited in **Figure** **6**a,b with the time‐voltage curves on the left, waterfall curves in the middle, and contour maps in rainbow color on the right. Although based on the galvanostatic charging/discharging curves the N‐NVP/N‐CN exhibits larger polarization. In general, both of them have shown a similar phase evolution fact by those peaks appearing at the same positions and similar electrochemical behaviors, following two steps of three‐phase redox reactions with two electrons transferred. However, the intermediate Na_2_V_2_(PO_4_)_3_ in NVP/N‐CB of the in situ XRD patterns are not well distinguished due to the apparent reaction lag, while the phase changes in N‐NVP/N‐CN electrode are clearly observed. The associated equations are presented as follows:

(1) $${\mathrm{Na}}_{3}V_{2}{\left({\mathrm{PO}}_{4}\right)}_{3}⇋{\mathrm{Na}}_{2}V_{2}{\left({\mathrm{PO}}_{4}\right)}_{3}+{\mathrm{Na}}^{+}+e^{-}$$

(2) $${\mathrm{Na}}_{2}V_{2}{\left({\mathrm{PO}}_{4}\right)}_{3}⇋{\mathrm{NaV}}_{2}{\left({\mathrm{PO}}_{4}\right)}_{3}+{\mathrm{Na}}^{+}+e^{-}$$

These are based on the apparent intermediate of Na_2_V_2_(PO_4_)_3_ (Space group of *P2_1_/c* with parameters of *a* = 15.41, *b* = 8.73, and *c* = 8.82, and typical peaks at 24.3°, 29.2°, 32.5°, etc.) as shown in the in situ XRD patterns. As charging, the peaks at ≈23.8°, 28.8°, and 32.1° of N‐NVP/N‐CN, referring to Na_3_V_2_(PO_4_)_3_, gradually disappeared in the first few patterns, and the intermediate of Na_2_V_2_(PO_4_)_3_ is generated clearly, leading to the space groups transferring from *R3c* to *P2*_1_/*c*. Then, the intermediate is successfully transferred into NaV_2_(PO_4_)_3_ with typical peaks at 24.6°, 29.7°, and 32.9° in the remaining patterns, resulting in the space groups transferring from *P2*_1_ */c* to *R3c*, illustrating the existence of Na_2_V_2_(PO_4_)_3_ is facilitating to reduce the lattice mismatch between Na_3_V_2_(PO_4_)_3_ and NaV_2_(PO_4_)_3_ for maintaining the fast phase transition and high ionic diffusion coefficient. More detailed peak positions of the three phases are shown in Figure S34a–c (Supporting Information), displaying apparent peak differences and solid evidence for the intermediate of Na_2_V_2_(PO_4_)_3_. When discharging, the two steps of three‐phase reactions are fully reversed with obvious peaks in different discharging stages, among them, the intermediate of Na_2_V_2_(PO_4_)_3_ is also clearly observed, especially at 29.2°. The intermediate Na_2_V_2_(PO_4_)_3_ in N‐NVP/N‐CN demonstrates smoother phase conversion due to fast ion and electron transfer rate. Besides, there are apparent differences in both the electrochemical behaviors and the phase features in peak intensity and peak width at half height (PWHH) of NVP/N‐CB and N‐NVP/N‐CN. Only one charging/discharging platform of NVP/N‐CB is observed, relating to the slow equilibrium feature and high electrode polarization. In contrast, two apparent charging/discharging platforms are shown in the time‐voltage curves of N‐NVP/N‐CN. Furthermore, the higher peak intensity and smaller PWHH of N‐NVP/N‐CN are detected, giving a fast phase transition, low inner polarization, and fast equilibrium feature, which are highly associated with the surface crystal modification by doping N in NVP for good conductivity and short Na^+^ ion paths. The current density during charging/discharging process will influence the exploration of the Na_2_V_2_(PO_4_)_3_ intermediate,^[^ ^25^ ^]^ which results in two voltage platforms in voltage‐time curve. Second, the stress of N‐NVP/N‐CN and NVP/N‐CB suffered during cycles are presented in Figure 6c, calculated via the Gauss equation analysis, largely revealing the stress on electrodes obeying the general Hooke's law by the proportional relationship. When charging, NVP/N‐CB and N‐NVP/N‐CN electrodes are both experiencing slight strains due to the extraction of Na^+^ ions and the shrinkage of volume cells. The stress of NVP/N‐CB is fluctuating at a relatively high value while the stress of N‐NVP/N‐CN is gradually decreasing to the lowest value and then recovering original state during charging/discharging progress because of the surface crystal modification by doping nitrogen and the porous feature, stemming from the lower inner stress and relatively stable structural stability.^[^ ^24^ ^]^ The stable structure was proved by the scanning electron microscopy (SEM) and TEM images after a long circulation (Figures S35 and S36, Supporting Information), providing a guarantee for the excellent cycling stability of N‐NVP/N‐CN. Therefore, the rationally constructed carbon nanocages encapsulated NVP nanoparticles, and then aroused the effect of gas chamber when pyrolysis of ammonium salt and PVP, enforcing the N‐doping effect by NH_3_ gas on the surface of NVP and carbon shell, thus largely decreasing the bandgap of N‐NVP/N‐CN but boosting its electrochemical performances. The strategy of structural modification and heteroatomic doping factually tune the sodium cation transportation, further promoting to the possibility of discovering the three‐phase reaction of Na_3_V_2_(PO_4_)_3_ – Na_2_V_2_(PO_4_)_3_ – Na_1_V_2_(PO_4_)_3_ system by in situ XRD and reducing the reaction hysteresis and reaction energy barriers during the electrochemical activities.

## 3 Conclusion

In conclusion, the scalable N‐NVP/N‐CN was synthesized by a spray‐drying approach followed by carbonization. During calcination, due to the EGC, abundant N atom is doped on the surface of NVP lattice (named N‐NVP) to decrease bandgap and energy barrier of Na^+^ diffusion kinetic from bulk to electrolyte or through bulk. Amazingly, the fast electron and ion transfer rate promote the formation of intermediate Na_2_V_2_(PO_4_)_3_ phase, which facilitates the fast reversible three‐phase reaction in Na_3_V_2_(PO_4_)_3_ – Na_2_V_2_(PO_4_)_3_ – Na_1_V_2_(PO_4_)_3_ system of N‐NVP. Simultaneously, compared to NVP/N‐CB and N‐NVP/N‐CP, the N‐NVP/N‐CN with a shrinking surface can provide a channel for permeation of electrolyte to increase the contacting area with the electrode material and shorten the diffusion path of ion and electron, which provides more active sites for the redox reaction to cast excellent rate capability. Consequently, as cathode materials for SIB, N‐NVP/N‐CN exhibits a high specific capacity of 119.7 mAh g^−1^ at 1 C after 100 cycles. Amazingly, high capacities of 89.0, 86.2, and 84.6 mAh g^−1^ are achieved after overlong 10 000 cycles at 20, 40, 50 C, respectively, which demonstrates the excellent cycling stability. This approach provides a new idea for surface crystal modification to cast intermediate Na_2_V_2_(PO_4_)_3_ phase for fast reversible electrochemical conversion of Na_3_V_2_(PO_4_)_3_, companying with microstructure modification to achieve excellent cycling stability and rate capability.

## 4 Experimental Section

### Synthesis of N‐NVP/N‐CN with shrinking surface

The NaAc, NH_4_VO_3,_ and NH_4_H_2_PO_4_ were dissolved into deionized water at a stoichiometric ratio of 3: 2: 3, and then 0.5 g Polyvinylpyrrolidone (PVP) and 600 mg polystyrene (PS) sphere were added to the above solution, which was put into spray dryer (XINW‐6000Y). Then the Na_3_V_2_(PO_4_)_3_/PVP precursor was synthesized by spray drying at a power of 80 Hz with a temperature of 180 °C. Finally, the surface N‐doped NVP encapsulating by N‐doped carbon nanocage (N‐NVP/N‐CN) was obtained after calcination at a temperature of 800 °C for 8 h in a tube furnace under N_2_ gas. The N‐doped NVP/N‐C hollow polyhedron (N‐NVP/N‐CP) was constructed by the same approach except for adding PS spheres, and the NVP/N‐C bulk (NVP/N‐CB) was constructed by a hydrothermal method.

### Characterization

The X‐ray diffraction (XRD) patterns were performed on a Panalytical X' Pert PRO MPD [Cu Ka radiation (*λ* = 1.5405 Å)], operating at 40 kV and 40 mA. Scanning electron microscopy (SEM) images were got by using a JSM‐6700 microscope operating at 5.0 kV. The transmission electron microscopy (TEM) images were obtained using JEM‐2100 (UHR) at 200 kV. The nitrogen adsorption–desorption isotherms were measured on a V‐Sorb 2800P (China Jinaipu) sorption analyzer under liquid nitrogen (−196 °C) with prior degassing under vacuum at 120 °C for >6 h. X‐ray photon spectroscopy (XPS) spectra were recorded by an ESCALAB 250 Xi XPS system of Thermo Scientific, where the analysis chamber was 1.5 × 10^−9^ mbar and the X‐ray spot was 500 µm. The XPS data were corrected regarding C1s (284.8 eV). The Raman spectra were performed by a HR‐800 (Jobin Yvon). The fourier transform infrared spectroscopy (FTIR) was tested by an ALPHA (German Bruker) instrument. The X‐ray absorption near‐edge structure (XANES) and extended X‐ray absorption fine structure (EXAFS) were performed at Beamlines 1W1B from Beijing Synchrotron Radiation Facility (BSRF) taking the transmission as modes.

### Electrochemical Characterization

Slurries of N‐NVP/N‐CN, N‐NVP/N‐CP, or NVP/N‐CB (70% w/w), super P (20% w/w), and PVDF binder (10% w/w) were mingled in a mortar for 20 min. The slurries were coated onto an aluminum foil and dried under vacuum for 8 h at 60 °C. The mass loading of active material in the electrode was 1.75 mg cm^−2^. The cell (model 2032) was fabricated using Na metal as the counter and reference electrode and contained an electrolyte mixture of 1.0 m NaClO_4_ dissolved in ethylene carbonate (EC) and dimethyl carbonate (DMC) (1:1 by volume) with 5% FEC. Galvanostatic measurements were made using a LAND CT2001A battery testing system. Cyclic voltammogram curves were examined with a voltage window between 2.5 and 3.8 V (vs Na/Na^+^) at different scan rates. Cyclability was determined by charging/discharging the cell at a range from 2.5 to 3.8 V at constant current rates of 1, 20, 40, 50, and 100 C. The capacity was calculated based on the mass of total NVP+C content. The rate capability was checked by a galvanostatic charge/discharge measurement at different current densities of 1, 2, 5, 10, 20, 40, 50, 100, 200, and 1 C, respectively. Electrochemical impedance spectroscopy (EIS) test was performed at a frequency range from 0.01 to 10^−5^ Hz with an ac amplification voltage of 10 mV.

### DFT Calculation

The density functional theory (DFT) calculations were performed using a Dmol3 module of Material Studio 2020. A surface model was cleaved from this supercell to simulate the (0 0 1) surface of the structure Na_3_V_2_(PO_4_)_3_. The generalized gradient approximation (GGA) method with Perdew–Burke–Ernzerhof (PBE) function was employed to describe the interactions between core and electrons. In the vertical direction, a vacuum layer of ≈10 Å in thickness was introduced for the surface. The force and energy convergence criterion were set to 0.002 Ha Å^−1^ and 10^−5^ Ha, respectively.

When the optimization was completed, the transition states were located utilizing the well‐known linear synchronous transit (LST) and quadratic synchronous transit (QST) methods. After the LST/QST calculations, the frequency calculations were performed. A true transition state from LST/QST calculations was confirmed by a single negative frequency. The free energy corrections were accomplished with dmol3 at a temperature of 298.15 K.

## Conflict of Interest

The authors declare no conflict of Interest.

## Supporting information

Supporting Information

## Data Availability

The data that support the findings of this study are available from the corresponding author upon reasonable request.

## References

[1] H. Kim , H. Kim , Z. Ding , M. H. Lee , K. Lim , G. Yoon , K. Kang , Adv. Energy Mater. 2016, 6, 1600943.

[2] R. Ling , S. Cai , D. Xie , X. Li , M. Wang , Y. Lin , S. Jiang , K. Shen , K. Xiong , X. Sun , Chem. Eng. J. 2018, 353, 264.

[3] S. Chu , S. Guo , H. Zhou , Chem. Soc. Rev. 2021, 50, 13189. 34719701 10.1039/d1cs00442e

[4] E. Wang , M. Chen , X. Liu , Y. Liu , H. Guo , Z. Wu , W. Xiang , B. Zhong , X. Guo , S. Chou , S.‐X. Dou , Small Methods 2019, 3, 1800169.

[5] X. Yao , Z. Zhu , Q. Li , X. Wang , X. Xu , J. Meng , W. Ren , X. Zhang , Y. Huang , L. Mai , ACS Appl. Mater. Interfaces 2018, 10, 10022. 29493210 10.1021/acsami.7b16901

[6] Z. Jian , C. Yuan , W. Han , X. Lu , L. Gu , X. Xi , Y.‐S. Hu , H. Li , W. Chen , D. Chen , Y. Ikuhara , L. Chen , Adv. Funct. Mater. 2014, 24, 4265.

[7] S. Sun , S. Liu , Y. Chen , L. Li , Q. Bai , Z. Tian , Q. Huang , Y. Wang , X. Wang , L. Guo , Adv. Funct. Mater. 2023, 33, 2213711.

[8] X.‐R. Qi , Y. Liu , L.‐L. Ma , B.‐X. Hou , H.‐W. Zhang , X.‐H. Li , Y.‐S. Wang , Y.‐Q. Hui , R.‐X. Wang , C.‐Y. Bai , H. Liu , J.‐J. Song , X.‐X. Zhao , Rare Met. 2022, 41, 1637.

[9] Q. Ni , Y. Bai , Y. Li , L. Ling , L. Li , G. Chen , Z. Wang , H. Ren , F. Wu , C. Wu , Small 2018, 14, 1702864. 10.1002/smll.201702864 29356385

[10] J. Mao , C. Luo , T. Gao , X. Fan , C. Wang , J. Mater. Chem. A 2015, 3, 10378.

[11] Y. Chen , Y. Xu , X. Sun , B. Zhang , S. He , L. Li , C. Wang , J. Power Sources 2018, 378, 423.

[12] Y. Li , X. Liang , G. Chen , W. Zhong , Q. Deng , F. Zheng , C. Yang , M. Liu , J. Hu , Chem. Eng. J. 2020, 387, 123952.

[13] W. Shen , C. Wang , Q. J. Xu , H. M. Liu , Y. G. Wang , Angew. Chem., Int. Ed. 2015, 5, 1400982.

[14] L. Zhao , H. Zhao , J. Wang , Y. Zhang , Z. Li , Z. Du , K. Swierczek , Y. Hou , ACS Appl. Mater. Interfaces 2021, 13, 8445. 33560822 10.1021/acsami.0c21861

[15] L. L. Ma , L. J. Yu , J. C. Liub , Y. Q. Su , S. Li , X. H. Zang , T. Meng , S. H. Zhang , J. J. Song , J. Y. Wang , X. X. Zhao , Z. M. Cui , N. Wang , Y. Zhao , Energy Storage Mater. 2022, 44, 180.

[16] L. Ma , B. Hou , N. Shang , S. Zhang , C. Wang , L. Zong , J. Song , J. Wang , X. Zhao , Mater. Chem. Front. 2021, 5, 4579.

[17] H. Xiong , R. Qian , Z. Liu , R. Zhang , G. Sun , B. Guo , F. Du , S. Song , Z.‐A. Qiao , S. Dai , Adv. Sci. 2021, 8, 2004943. 10.1002/advs.202004943 PMC8188202 34105293

[18] X. Tang , C. Liu , H. Wang , L.‐P. Lv , W. Sun , Y. Wang , Coord. Chem. Rev. 2023, 494, 215361.

[19] S. Kajiyama , J. Kikkawa , J. Hoshino , M. Okubo , E. Hosono , Chem. ‐ Eur. J. 2014, 20, 12636. 25123497 10.1002/chem.201403126

[20] S. Gao , N. Wang , S. Li , D. Li , Z. Cui , G. Yue , J. Liu , X. Zhao , L. Jiang , Y. Zhao , Angew. Chem., Int. Ed. 2020, 59, 2465. 10.1002/anie.201913170 31788929

[21] X. Zhao , J. Wang , R. Yu , D. Wang , J. Am. Chem. Soc. 2018, 140, 17114. 30428662 10.1021/jacs.8b09241

[22] B. Hou , L. Ma , X. Zang , N. Shang , J. Song , X. Zhao , C. Wang , J. Qi , J. Wang , R. Yu , Chem. Res. Chin. Univ. 2021, 37, 265.

[23] L. Liang , X. Li , F. Zhao , J. Zhang , Y. Liu , L. Hou , C. Yuan , Adv. Eng. Mater. 2021, 11, 2100287.

[24] Z. Wang , J. Liu , Z. Du , H. Tao , Y. Yue , Inorg. Chem. Front. 2020, 7, 1289.

[25] S. Park , Z. L. Wang , Z. Y. Deng , I. Moog , P. Canepa , F. Fauth , D. Carlier , L. Croguennec , C. Masquelier , J. N. Chotard , Chem. Mater 2022,34, 451.

[26] C. Wang , J. Wang , W. Hu , D. Wang , Chem. Res. Chin. Univ. 2020, 36, 68.

[27] J. Zhao , M. Yang , N. Yang , J. Wang , D. Wang , Chem. Res. Chin. Univ. 2020, 36, 313.

[28] C. Liu , Z.‐X. Zhang , R. Tan , J.‐W. Deng , Q.‐H. Li , X.‐C. Duan , Rare Met. 2021, 41, 806.

[29] X.‐X. Gu , S. Qiao , X.‐L. Ren , X.‐Y. Liu , Y.‐Z. He , X.‐T. Liu , T.‐F. Liu , Rare Met. 2021, 40, 828.

[30] L. Xu , J. Li , Y. Li , P. Cai , C. Liu , G. Zou , H. Hou , L. Huang , X. Ji , Chem. Res. Chin. Univ. 2020, 36, 459.

[31] B. Guo , W. Du , T. Yang , J. Deng , D. Liu , Y. Qi , J. Jiang , S.‐J. Bao , M. Xu , Adv. Sci. 2020, 7, 1902617. 10.1002/advs.201902617 PMC7029643 32099760

[32] W. Choi , R. K. Bera , S. W. Han , H. Park , T. W. Go , M. Choi , R. Ryoo , J. Y. Park , Carbon 2022, 193,42.

[33] C. Huang , L. Yu , W. Zhang , Q. Xiao , J. Zhou , Y. Zhang , P. An , J. Zhang , Y. Yu , Appl. Catal. B 2020, 276, 119137.

[34] T.‐F. Hung , W.‐J. Cheng , W.‐S. Chang , C.‐C. Yang , C.‐C. Shen , Y.‐L. Kuo , Chem. ‐ Eur. J. 2016, 22, 10620. 27346677 10.1002/chem.201602066

[35] D. Yu , Q. Pang , Y. Gao , Y. Wei , C. Wang , G. Chen , F. Du , Energy Storage Mater. 2018, 11, 1.

[36] G. D. Yuan , Z. Z. Ye , J. Y. Huang , Z. P. Zhu , C. L. Perkins , S. B. Zhang , J. Cryst. Growth 2009, 311, 2341.

[37] T. Meng , J. Qin , Z. Yang , L. Zheng , M. Cao , J. Mater. Chem. A 2019, 7, 17570.

[38] M. Chen , W. Hua , J. Xiao , D. Cortie , X. Guo , E. Wang , Q. Gu , Z. Hu , S. Indris , X.‐L. Wang , S.‐L. Chou , S.‐X. Dou , Angew. Chem., Int. Ed. 2020, 132, 2470. 10.1002/anie.201912964 31657087

[39] L. Zhang , N. Shang , S. Gao , J. Wang , T. Meng , C. Du , T. Shen , J. Huang , Q. Wu , H. Wang , Y. Qiao , C. Wang , Y. Gao , Z. Wang , ACS Catal. 2020, 10, 8672.

[40] J. Cao , Y. Wang , L. Wang , F. Yu , J. Ma , Nano Lett. 2019, 19, 823. 30658040 10.1021/acs.nanolett.8b04006

[41] X. Liu , E. Wang , G. Feng , Z. Wu , W. Xiang , X. Guo , J. Li , B. Zhong , Z. Zheng , Electrochim. Acta 2018, 286, 231.

[42] Y. H. Jung , C. H. Lim , D. K. Kim , J. Mater. Chem. A 2013, 1, 11350.

[43] G.‐D. Yi , C.‐L. Fan , Z. Hu , W.‐H. Zhang , S.‐C. Han , J.‐S. Liu , Electrochim. Acta 2021, 383, 138370.

[44] C. Wu , H. Song , C. Tang , A. Du , C. Yu , Z. Huang , M. Wu , H. Zhang , Chem. Eng. J. 2019, 378, 122249.

[45] M. Li , Y. Zhu , X. Wu , Y. Lei , X. He , Q. Li , R. Jiang , Z. Lei , Z. Liu , J. Sun , ACS Appl. Energy Mater. 2021, 4, 5713.

[46] Y. Cao , H. Fang , C. Guo , W. Sun , Y. Xu , Y. Wu , Y. Wang , Angew. Chem., Int. Ed. 2023, 62, e202302143. 10.1002/anie.202302143 37269463

[47] H. Wang , W. Zou , C. Liu , Y. Sun , Y. Xu , W. Sun , Y. Wang , Batteries Supercaps 2023, 6, 202200434.

[48] H. Zhang , I. Hasa , D. Buchholz , B. Qin , S. Passerini , Carbon 2017, 124, 334.

[49] W. Li , Z. J. Yao , C. A. Zhou , X. L. Wang , X. H. Xia , C. D. Gu , J. P. Tu , Small 2019, 15, 190243.

[50] X. Liang , X. Ou , F. Zheng , Q. Pan , X. Xiong , R. Hu , C. Yang , M. Liu , ACS Appl. Mater. Interfaces 2017, 9, 13151. 28345855 10.1021/acsami.7b00818

[51] A. Sadezky , H. Muckenhuber , H. Grothe , R. Niessner , U. Pöschl , Carbon 2005, 43, 1731.

[52] K. Cui , S. Hu , Y. Li , Electrochim. Acta 2016, 210, 45.

[53] L. B. Zong , W. C. Wu , S. L. Liu , H. J. Yin , Y. A. Chen , C. Liu , K. C. Fan , X. X. Zhao , X. Chen , F. M. Wang , Y. Yang , L. Wang , S. H. Feng , Energy Storage Mater. 2020, 27, 514.

[54] J. Xu , E. Gu , Z. Zhang , Z. Xu , Y. Xu , Y. Du , X. Zhu , X. Zhou , J. Colloid Interface Sci. 2020, 567, 84. 32036117 10.1016/j.jcis.2020.01.121

[55] H. Zhang , P. Zuo , J. Hua , Y. Ma , C. Du , X. Cheng , Y. Gao , G. Yin , Electrochim. Acta 2017,238, 257.

[56] J. F. Yang , D. D. Li , X. S. Wang , X. X. Zhang , J. Xu , J. T. Chen , Energy Storage Mater. 2020, 24, 694.

[57] T. Wei , G. Yang , C. Wang , Nano Energy 2017, 39, 363.

[58] S. Li , P. Ge , C. Zhang , W. Sun , H. Hou , X. Ji , J. Power Sources 2017, 366, 249.

[59] M. K. Sadan , M. Jeon , J. Yun , E. Song , K.‐K. Cho , J.‐H. Ahn , H.‐J. Ahn , Sustainable Energy Fuels 2022, 6, 2155.

[60] Y. Zhou , X. Rui , W. Sun , Z. Xu , Y. Zhou , W. J. Ng , Q. Yan , E. Fong , ACS Nano 2015, 9, 4628. 25858505 10.1021/acsnano.5b00932

[61] X. Zhang , X. Rui , D. Chen , H. Tan , D. Yang , S. Huang , Y. Yu , Nanoscale 2019, 11, 2556. 30672554 10.1039/c8nr09391a

[62] H.‐B. Huang , S.‐H. Luo , C.‐L. Liu , Y. Yang , Y.‐C. Zhai , L.‐J. Chang , M.‐Q. Li , Appl. Surf. Sci. 2019, 487, 1159.

[63] Y. J. Fang , L. F. Xiao , X. P. Ai , Y. L. Cao , H. X. Yang , Adv. Mater. 2015, 27, 5859. 10.1002/adma.201502018 26305519

[64] X. Cao , A. Pan , B. Yin , G. Fang , Y. Wang , X. Kong , T. Zhu , J. Zhou , G. Cao , S. Liang , Nano Energy 2019, 60, 312.

[65] J. Li , Y. Chen , T. Zhou , H. Shi , Z. Zheng , Y. Wang , L. Guo , Appl. Surf. Sci. 2023, 610, 155553.

[66] B. Papandrea , X. Xu , Y. Xu , C.‐Y. Chen , Z. Lin , G. Wang , Y. Luo , M. Liu , Y. Huang , L. Mai , X. Duan , Nano Res. 2019, 9, 240.

[67] W. Li , D. Wang , Z. Gong , Z. Yin , X. Guo , J. Liu , C. Mao , Z. Zhang , G. Li , ACS Nano 2020, 14, 16046. 33147943 10.1021/acsnano.0c07733

[68] D. Sun , D. Ye , P. Liu , Y. Tang , J. Guo , L. Wang , H. Wang , Adv. Energy Mater. 2018, 8, 1702383.

[69] Y. Li , J. Qian , M. Zhang , S. Wang , Z. Wang , M. Li , Y. Bai , Q. An , H. Xu , F. Wu , L. Mai , C. Wu , Adv. Mater. 2020, 32, 2005802. 10.1002/adma.202005802 33089951

[70] B. Wang , Y. Ren , Y. Zhu , S. Chen , S. Chang , X. Zhou , P. Wang , H. Sun , X. Meng , S. Tang , Adv. Sci. 2023, 10, 2300860. 10.1002/advs.202300860 PMC10323615 37078796

[71] J. Ge , L. Fan , J. Wang , Q. Zhang , Z. Liu , E. Zhang , Q. Liu , X. Yu , B. Lu , Adv. Energy Mater. 2018, 8, 1801477.

