# Supplementary material for: Surface Crystal Modification of Na3V2(PO4)3 to Cast Intermediate Na2V2(PO4)3 Phase toward High‐Rate Sodium Storage
Source: Adv Sci (Weinh). 2023 Nov 23;11(3):2306168. doi: 10.1002/advs.202306168 (PMC10797425; doi:10.1002/advs.202306168)
Supplement: Supplementary file 1 — Supporting Information [file ADVS-11-2306168-s001.pdf]

ADVANCED SCIENCE
Open Access

Supporting Information

for *Adv. Sci.*, DOI 10.1002/advs.202306168

Surface Crystal Modification of $Na_3V_2(PO_4)_3$ to Cast Intermediate $Na_2V_2(PO_4)_3$ Phase toward High-Rate Sodium Storage

Hui Zhang, Lei Wang, Linlin Ma, Yahui Liu, Baoxiu Hou, Ningzhao Shang, Shuaihua Zhang, Jianjun Song, Shuangqiang Chen* and Xiaoxian Zhao**

Supporting Information

Surface crystal modification of $Na_3V_2(PO_4)_3$ to cast intermediate $Na_2V_2(PO_4)_3$ phase towards high-rate sodium storage

Hui Zhang[a‡], Lei Wang[c‡], Linlin Ma[a], Yahui Liu[d], Baoxiu Hou[a], Ningzhao Shang[a], Shuaihua Zhang[a], Jianjun Song[b], Shuangqiang Chen[c*], Xiaoxian Zhao[a*]*

[a] *Department of Chemistry, College of Science, Hebei Agricultural University, Baoding, 071001, China.*

[b] *College of Physics, Qingdao University, Qingdao 266071, P. R. China.*

[c] *Department of Chemical Engineering, School of Environmental and Chemical Engineering, Shanghai University, Shanghai 200444, P. R. China*

[d] *National Engineering Research Center of green recycling for strategic metal resources, Institute of Process Engineering, Chinese Academy of Sciences, Beijing 100190, PR China.*

‡ *These two authors contributed equally to this work.*

Corresponding author: E-mail: lxzhxx@hebau.edu.cn (X. Zhao); chensq@shu.edu.cn (S. Chen); jianjun.song@qdu.edu.cn (J. Song)

Supplemental Figures and Tables:

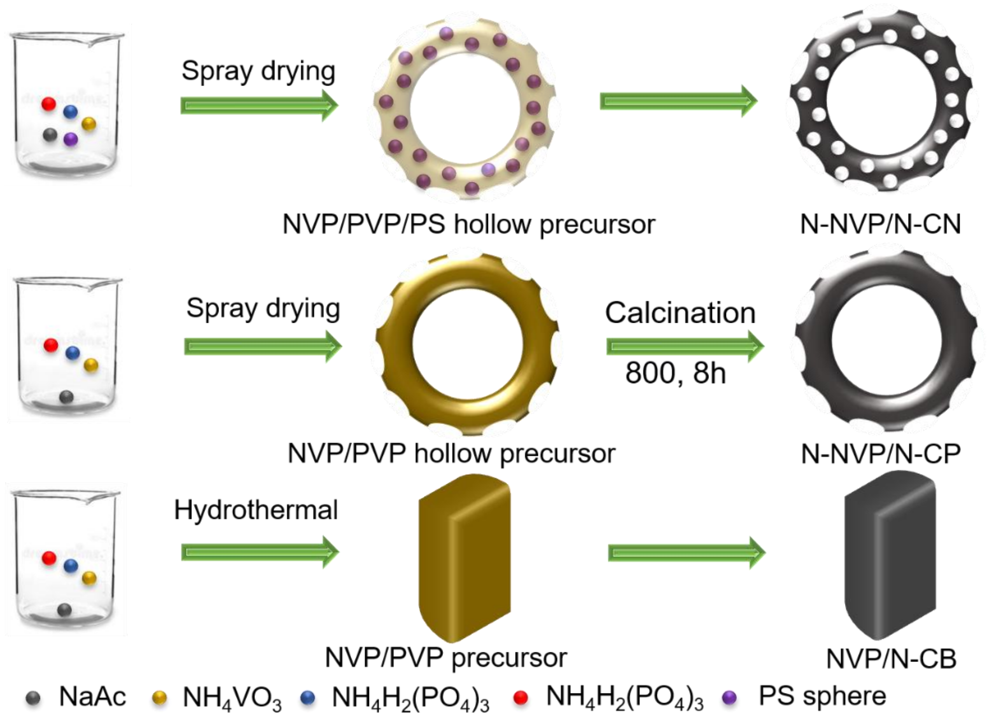

Figure S1. The preparation flow diagram of N-NVP/N-CN, N-NVP/N-CP, and NVP/N-CB.

The fabrication of N-NVP/N-CN involves the dissolution of NaAc, NH_4VO_3, and $(NH_4)_2H(PO_4)_3$ with a molar ratio of 3: 2: 3 in an aqueous solution containing polyvinyl pyrrolidone (PVP) and PS sphere, followed by spray drying and simple calcination process as illustrated in Fig. S1. Taken as contrast, the N-doped NVP/N-C hollow polyhedron (N-NVP/N-CP) was constructed by the same approach except adding PS spheres, and the NVP/N-C bulk (NVP/N-CB) was synthesized by a hydrothermal method.

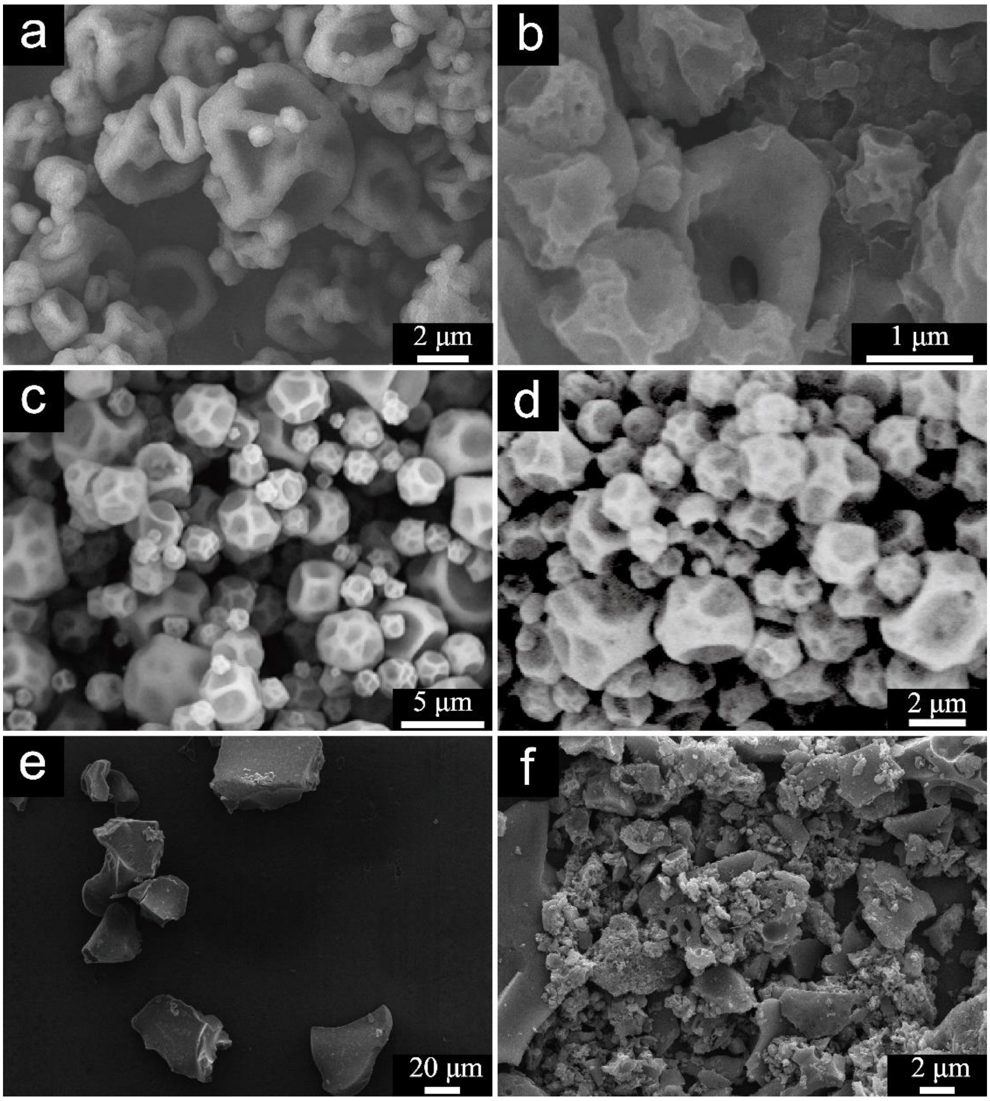

Figure S2. The SEM images of NVP/PVP precursor (a, N-NVP/N-CN; c, N-NVP/N-CP; e, NVP/N-CB) and product after calcination (b, N-NVP/N-CN; d, N-NVP/N-CP; f, NVP/N-CB).

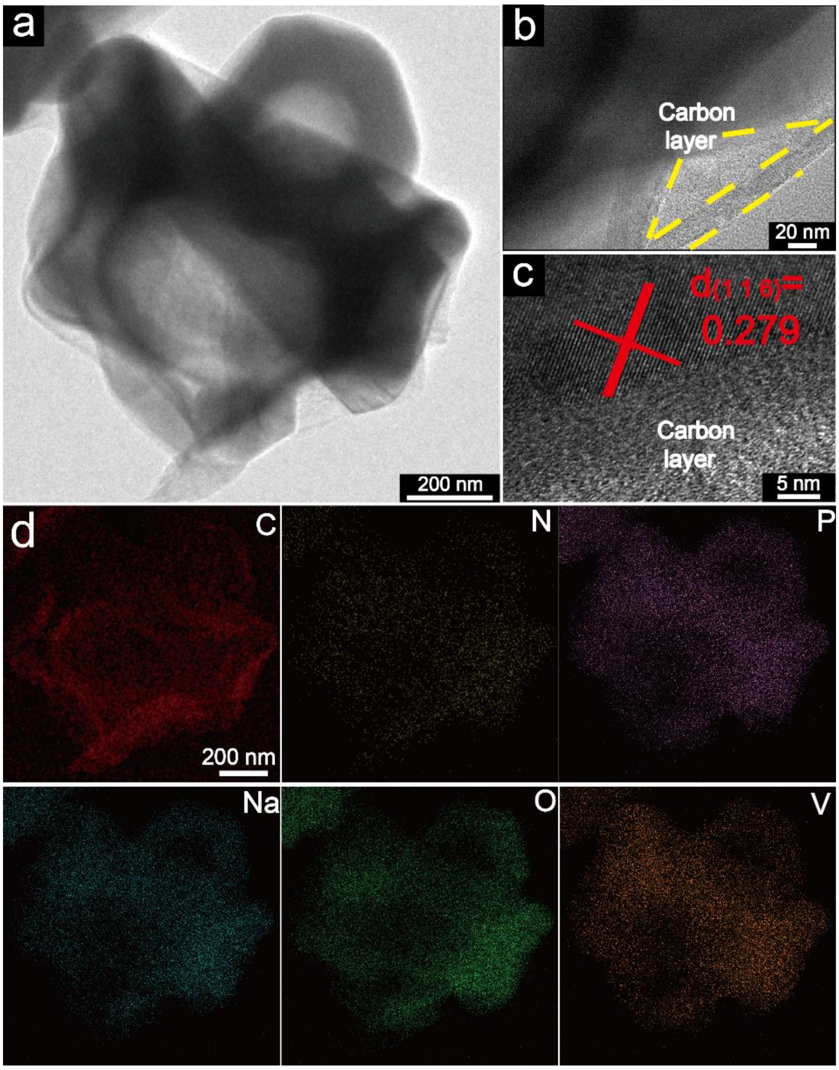

Figure S3. The TEM image (a), enlarged TEM image (b), HRTEM (c), and element mapping distribution (d) of N-NVP/N-CP.

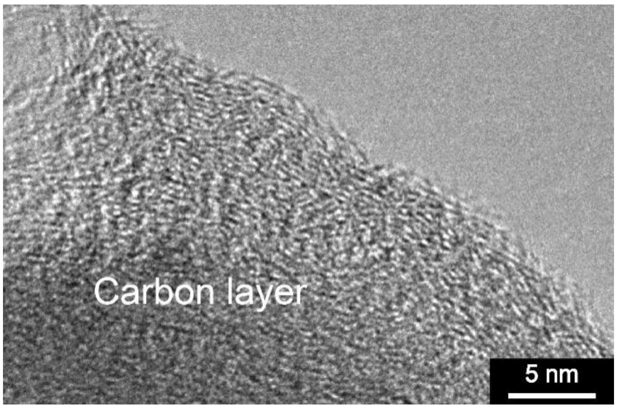

Figure S4. The HRTEM of carbon layer in N-NVP/N-CN.

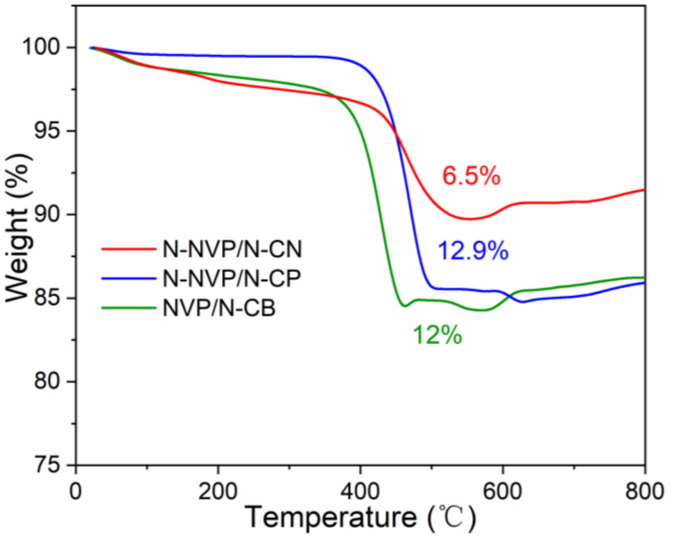

Figure S5. The TGA curves of N-NVP/N-CN, N-NVP/N-CP, and NVP/N-CB.

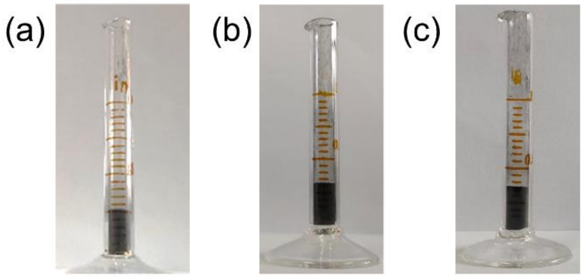

Figure S6. The tap density of N-NVP/N-CN, N-NVP/N-CP and NVP/N-CB.

(a) N-NVP/N-CN: 0.327g/0.26cm^3=1.26 g.cm^{-3}

(b) N-NVP/N-CP: 0.354g/0.30 cm^3=1.18 g.cm^{-3}

(c) NVP/N-CB: 0.469g/0.35 cm^3=1.34 g.cm^{-3}

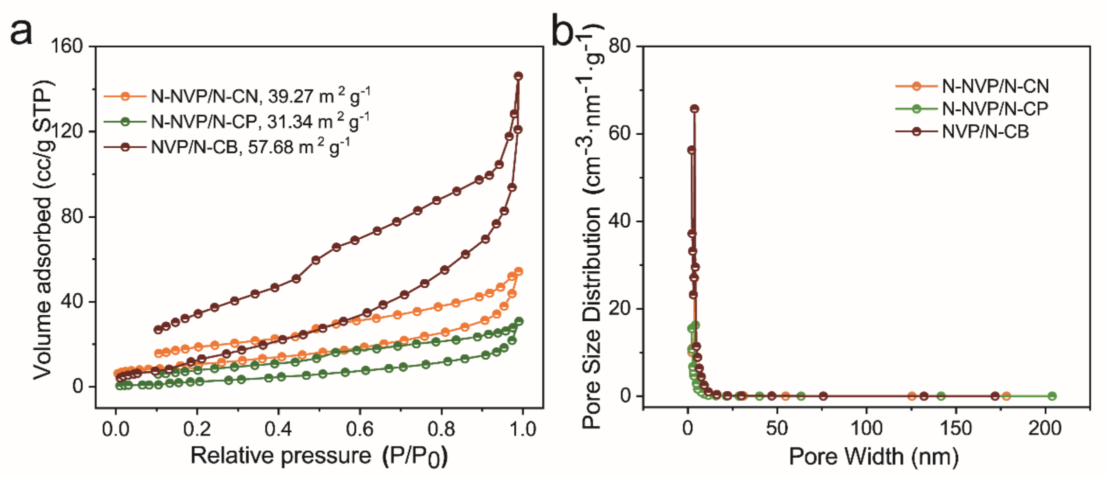

Figure S7. (a) N_2 adsorption/desorption isotherms and (b) pore size distribution of three materials (N-NVP/N-CN, N-NVP/N-CP, NVP-N-CB).

As shown in Figure S7a, the nitrogen adsorption/desorption isotherms of N-NVP/N-CN, N-NVP/N-CP and NVP/N-CB exhibit the specific surface areas are 39.27 m^2 g^{-1}, 31.34 m^2 g^{-1} and 57.68 m^2 g^{-1}, respectively. Although NVP/N-CB exhibits a larger specific surface area, N-NVP/N-CN has a larger effective active area. Besides, in Figure S7b, the pore size distribution shows that the pore types of N-NVP/N-CN are micropore and mesoporous.

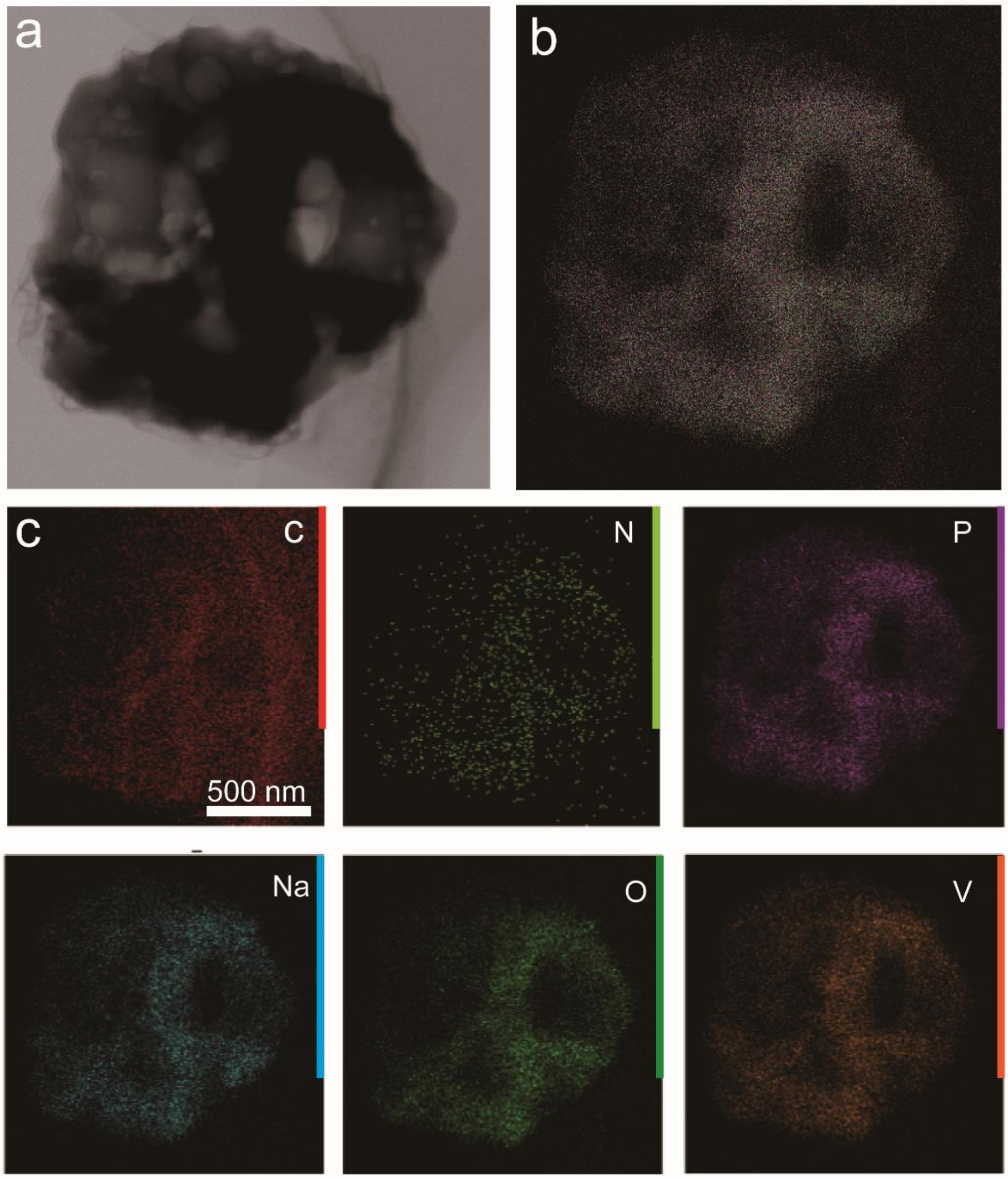

Figure S8. The element mapping distribution by TEM of N-NVP/N-CN.

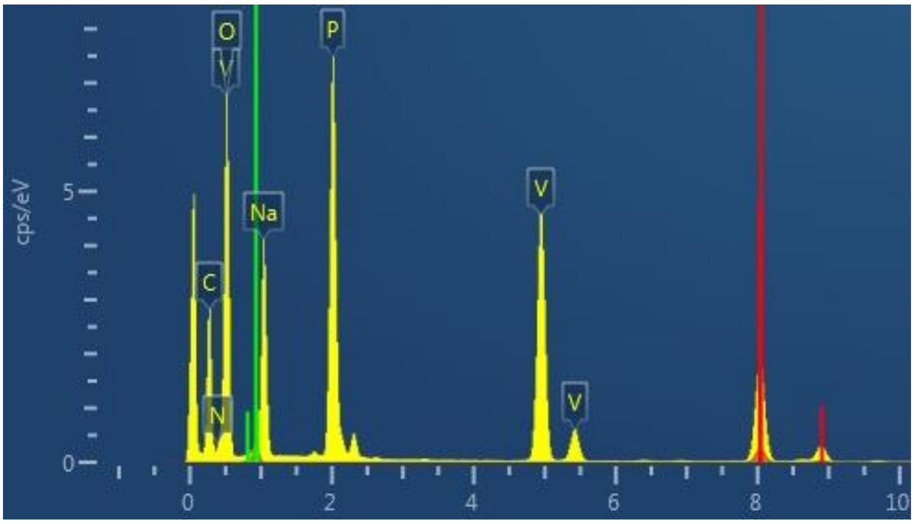

Figure S9. The energy spectrum analysis of N-NVP/N-CN.

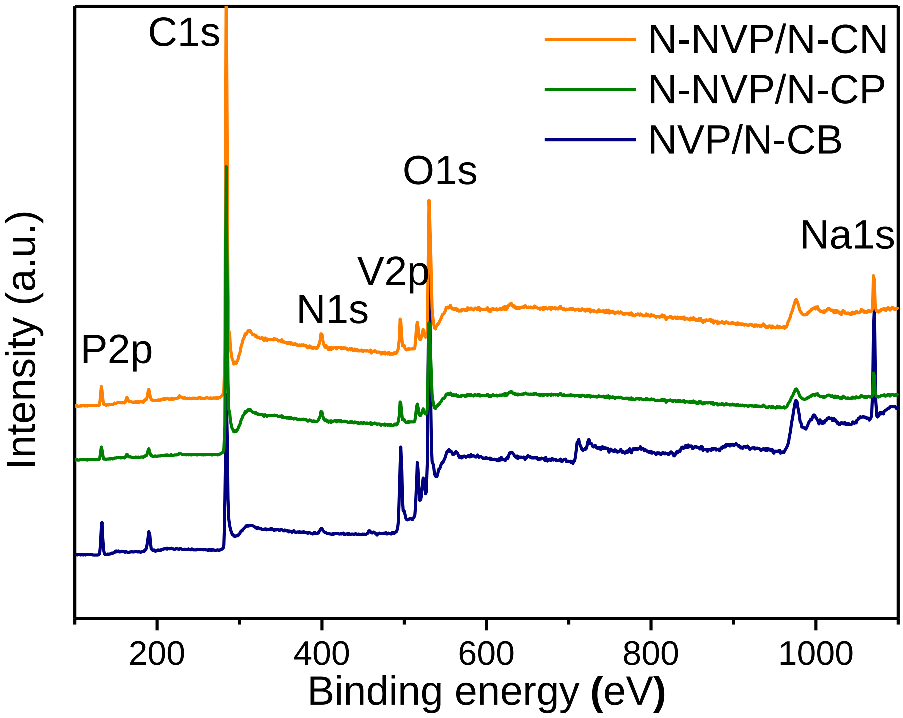

Figure S10. The XPS survey of N-NVP/N-CN, N-NVP/N-CP, and NVP/N-CB.

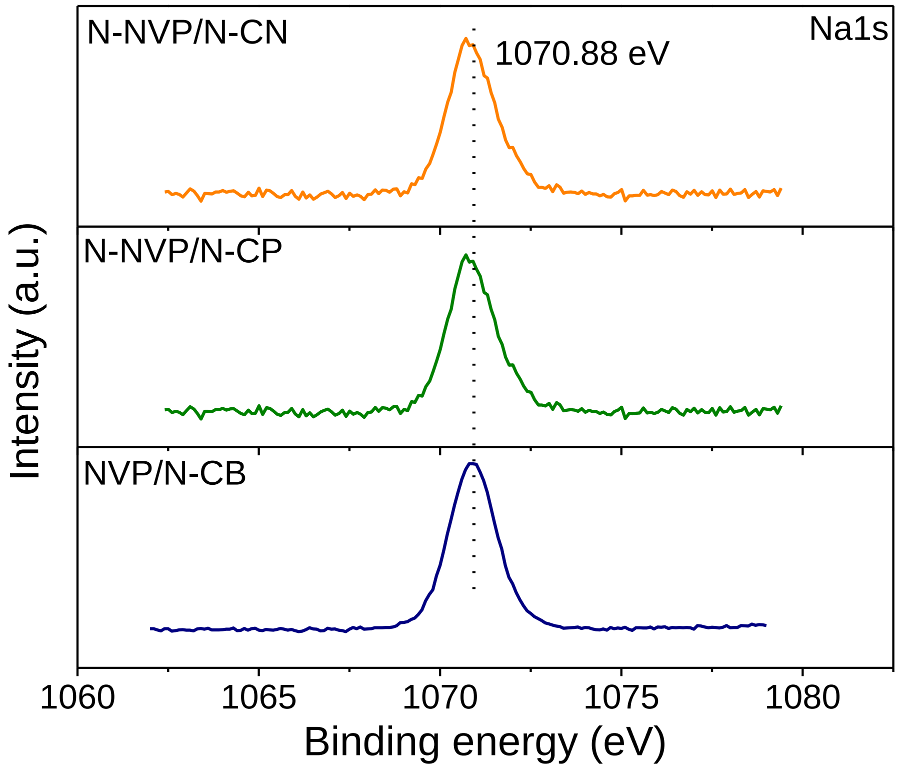

Figure S11. The Na1s high-resolution XPS analysis of N-NVP/N-CN, N-NVP/N-CP, and NVP/N-CB.

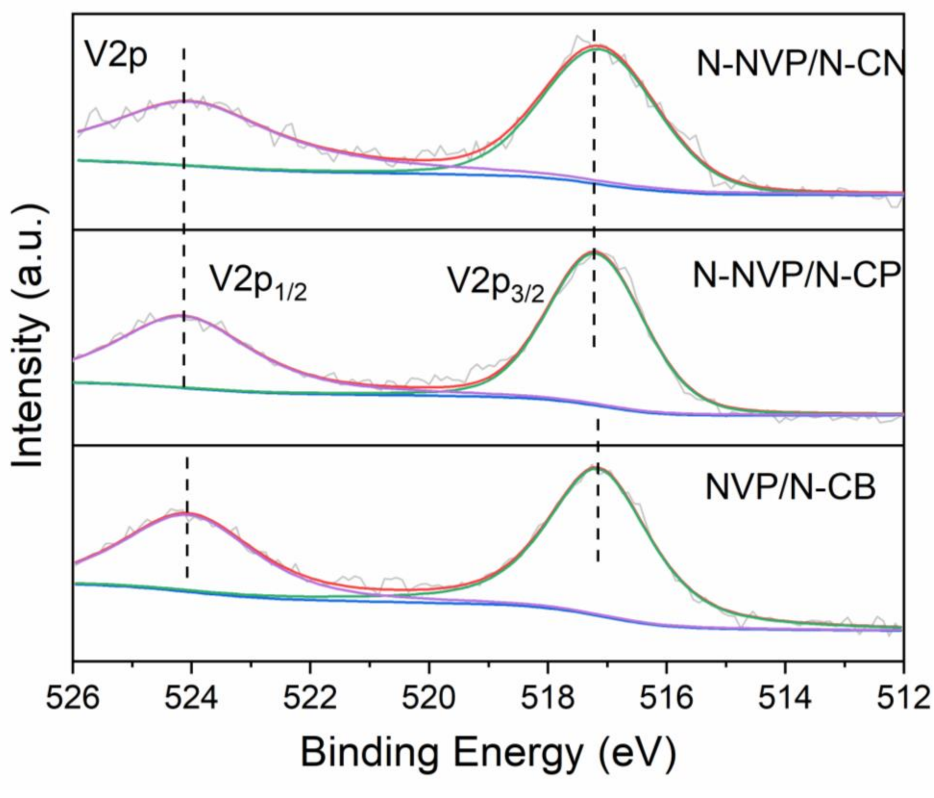

Figure S12. The V2p high-resolution XPS analysis of N-NVP/N-CN, N-NVP/N-CP, and NVP/N-CB.

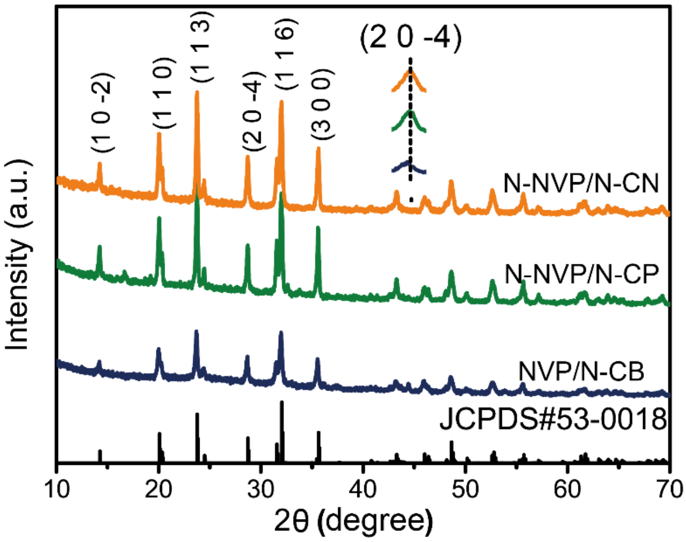

Figure S13. The XRD patterns of N-NVP/N-CN, N-NVP/N-CP, and NVP/N-CB.

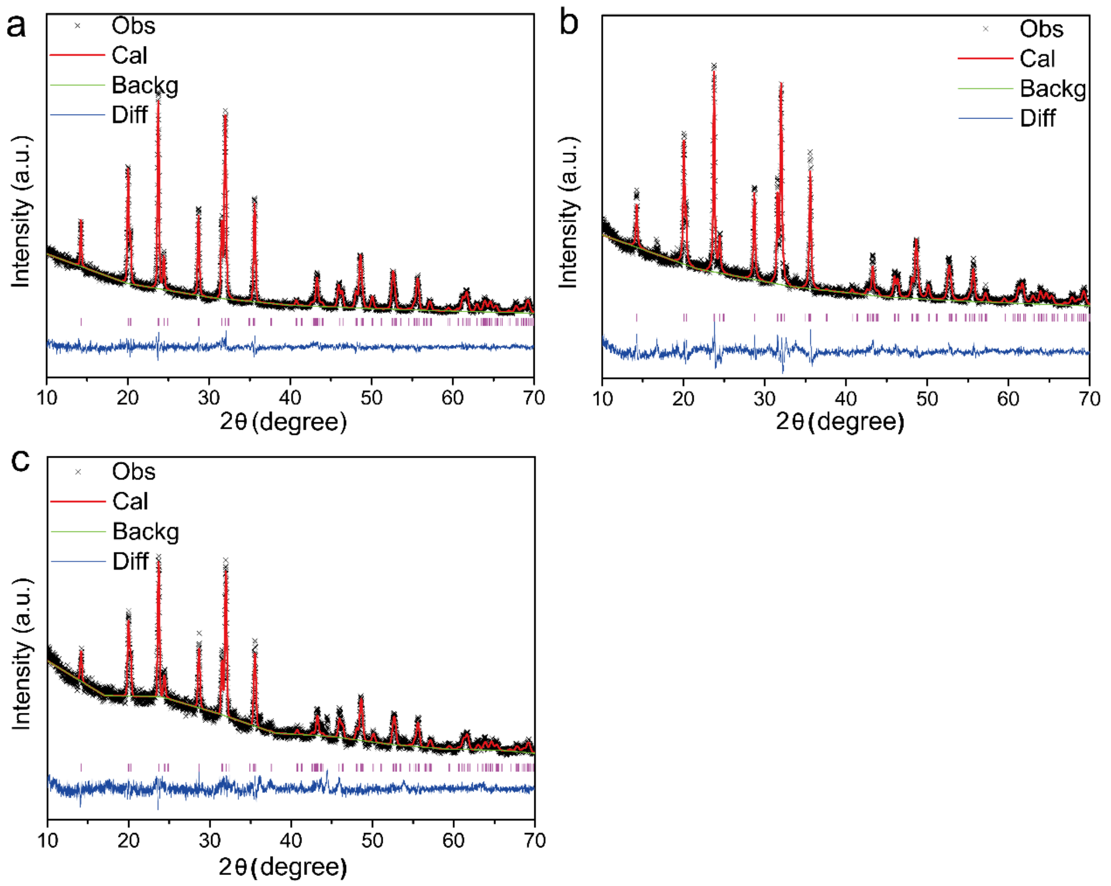

Figure S14. The XRD refinement curves of N-NVP/N-CN (a), N-NVP/N-CP(b) and NVP/N-CB (c).

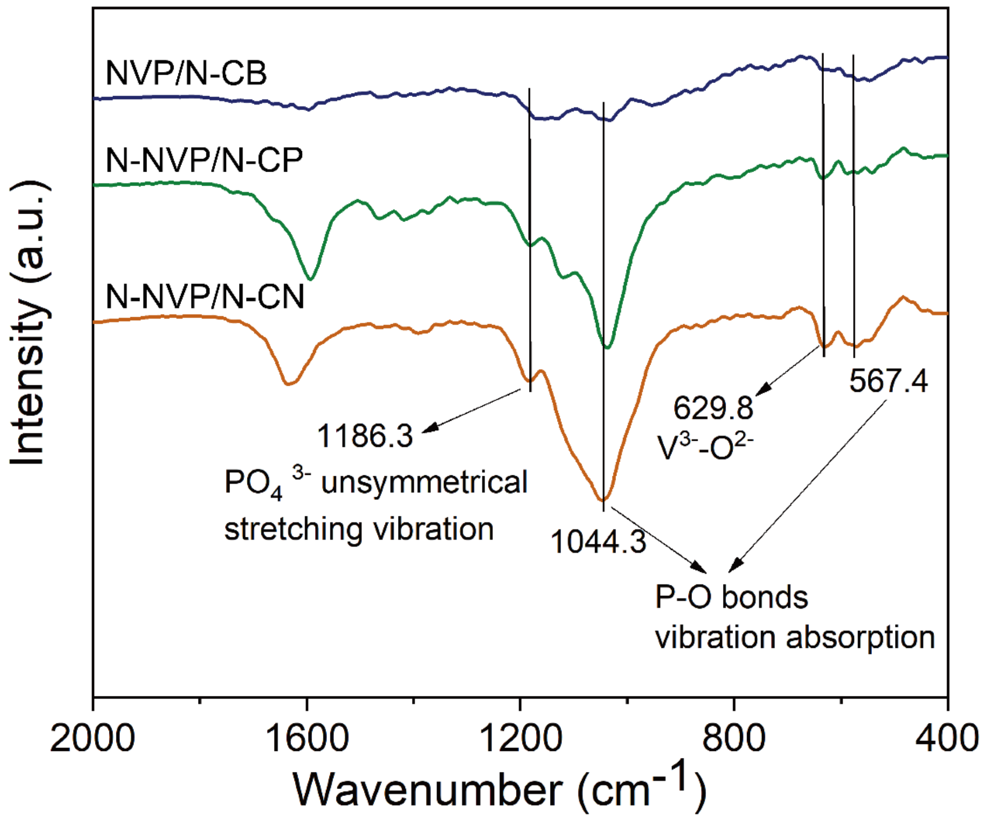

Figure S15. The FTIR curves of N-NVP/N-CN, N-NVP/N-CP, and NVP/N-CB.

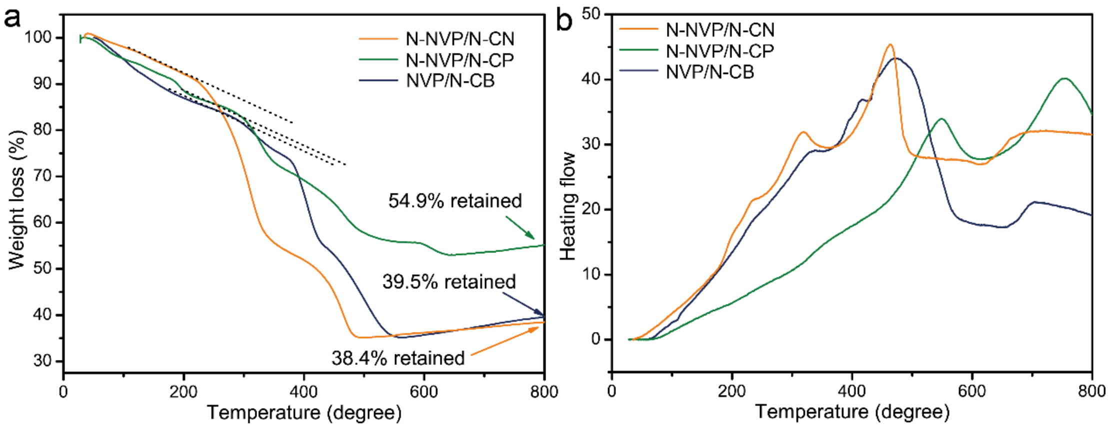

Figure S16. The Thermogravimetric Analysis (TGA) (a) and derivative thermogravimetric analysis (DTG) (b) curves of the precursors of N-NVP/N-CN, N-NVP/N-CP, and NVP/N-CB.

Due to the effect of the "nano gas chamber", the NH_3 generated from pyrolysis of ammonium salt precursor is harder to escape from the enclosed system even at high

temperature. Therefore, compared to NVP/N-CB, the rate of weight loss of N-NVP/N-CP is slow and the mass retained after 800 °C is higher. There are PS spheres in N-NVP/N-CN precursor, during calcination the PS spheres will decompose which leads to lower mass retained after 800 °C. However, the mass retained of N-NVP/N-CN is still similar to NVP/N-CB, which gives side evidence that the rate of weight loss of N-NVP/N-CN is slower than NVP/N-CB due to the effect of "nano gas chamber".

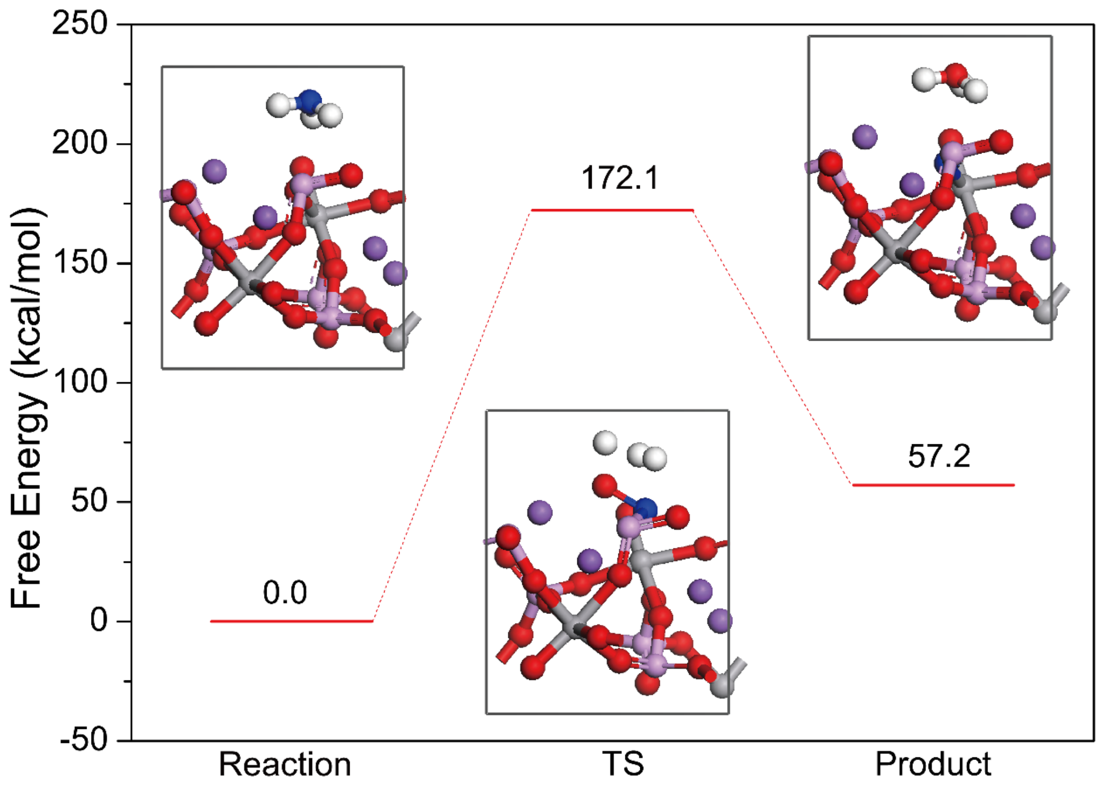

Figure S17. The diffusion barriers at various transition states from NVP to N-NVP through the reaction between O in phosphor oxygen hexahedron of NVP with NH_3.

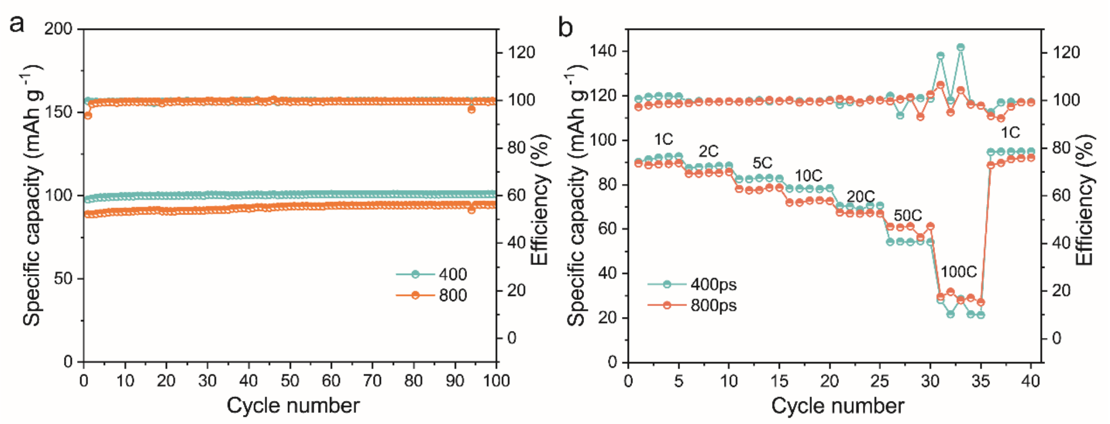

Figure S18. The cycling stability (a) and rate capability (b) of N-NVP/N-CN with different ratio of PVP and PS.

The cycling stability and rate capability with different PS spheres (400 mg, 600 mg and 800 mg corresponding to the PVP: PS ratio of 5: 4, 5: 6 and 5: 8) content was explored. As shown in Fig. S18a and S19b, materials prepared at different of PVP: PS spheres ratio shows the similar initial specific capacity at the current density of 1 C. However, as shwon in Fig. S18b and 4b, the N-NVP/N-CN (with 600 mg PS spheres) and the sample with 800 mg PS spheres demonstrate the higher specific capacity at high current densities. During calcination process, the PVP can be carbonized at high temperature in a N_2 gas, but the added PS spheres can be decomposed at high temperature to form porous structure. Therefore, the adding mass of PS spheres can influence the pore volume inside of the materials, which plays a key role in Na^+ diffusion kinetic. With increase of PS spheres, the pore volume inside of the material is increased, the providing active sites for redox reaction increases, which improves the rate capability. However, the more PS spheres added, the more pore volume inside, which decreases the energy density per unite volume. When the adding mass of PS sphere is 600 mg, the material can exsibite fast Na^+ diffusion kinetic and high volume density. Therefore, the adding mass of PS spheres is set as 600 mg.

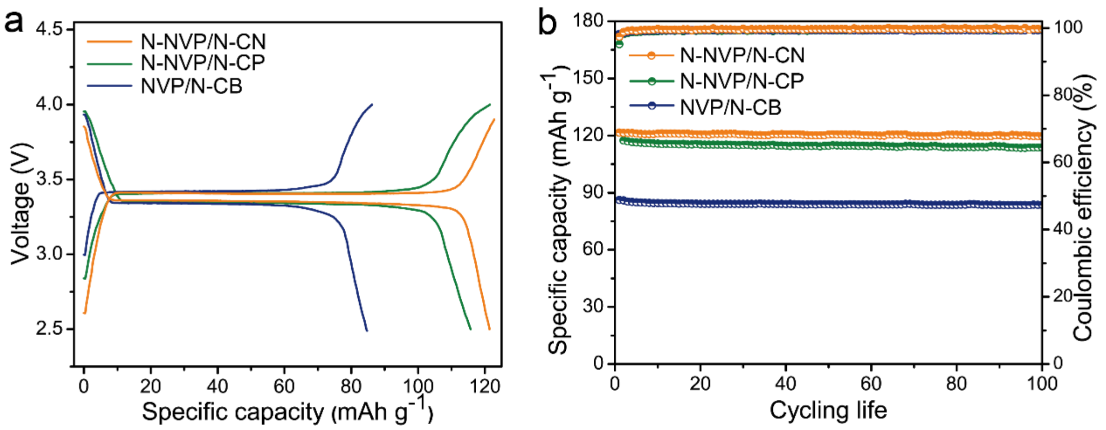

Figure S19. The initial charge/discharge curve (a) and the cycling stability at 1 C (b) of N-NVP/N-CN, N-NVP/N-CP, and NVP/N-CB.

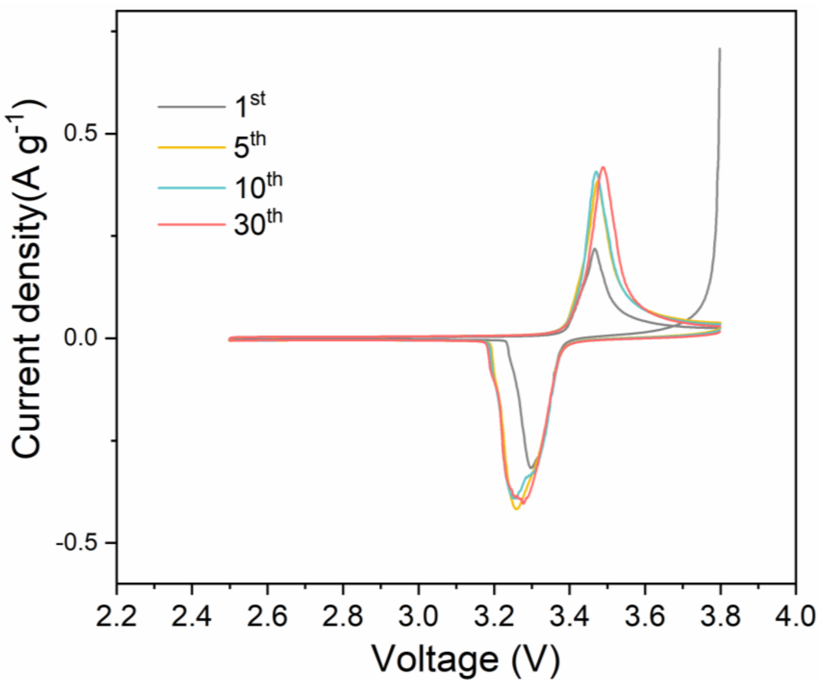

Figure S20. The cyclic voltammetry curves at a scan rate of 0.2 mV s^{-1} for 1st, 5th, 10th and 30th cycles in a voltage window of 2.5-3.8 V of N-NVP/N-CN.

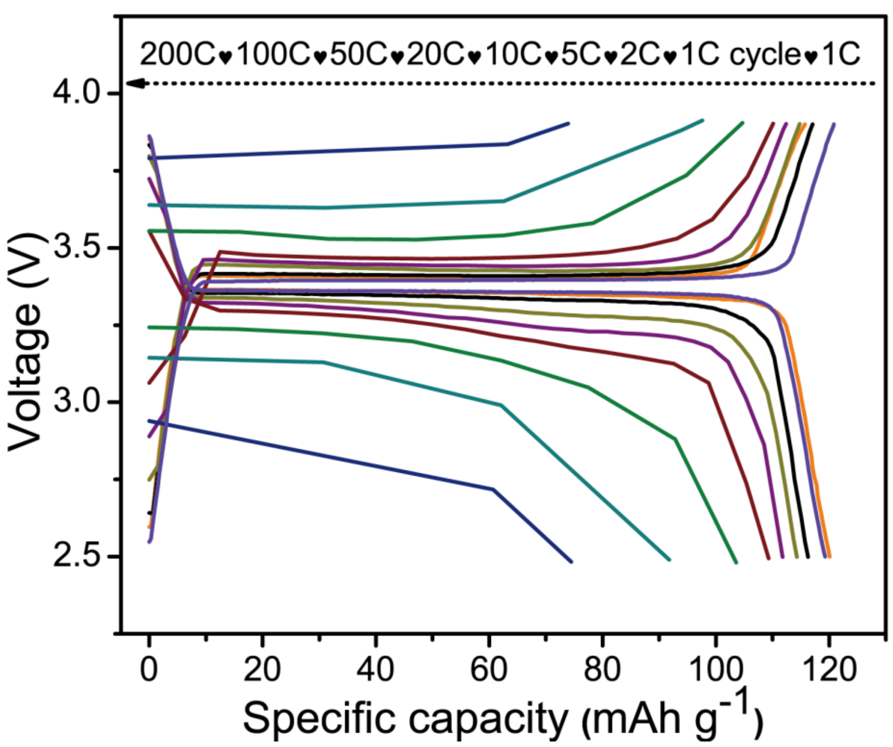

Figure S21. The galvanostatic charging/discharging curves at different current densities of 1 C, 2 C, 5 C, 10 C, 20 C, 50 C, 100 C, 200 C, and 1 C of N-NVP/N-CN, respectively.

Simultaneously, with the increase in current density, the charging and discharging platform increased and decreased slightly as shown in Fig. S21, respectively. A long platform can be detected even at a high current density of 200 C, which further proved the excellent rate capability of N-NVP/N-CN.

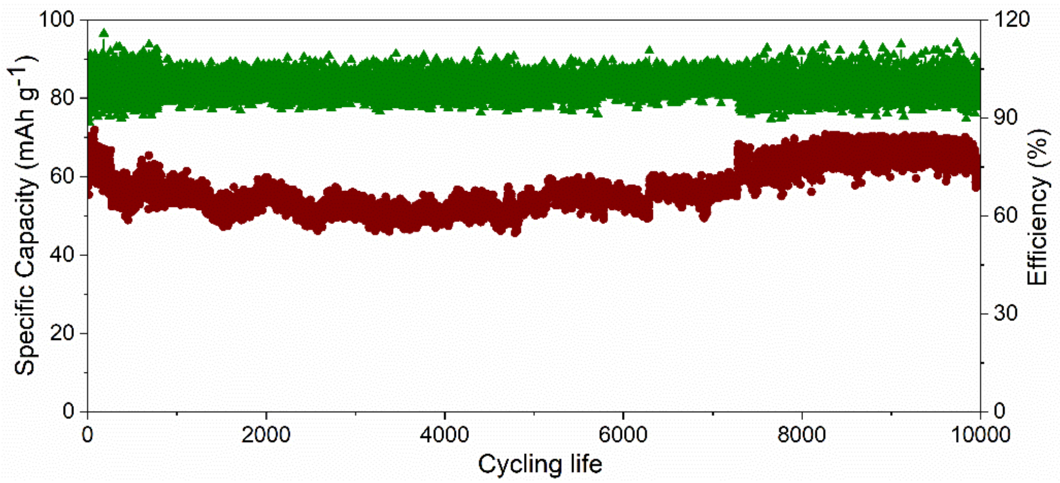

Figure S22. The long cycling stability of N-NVP/N-CN at high current densities of 100 C.

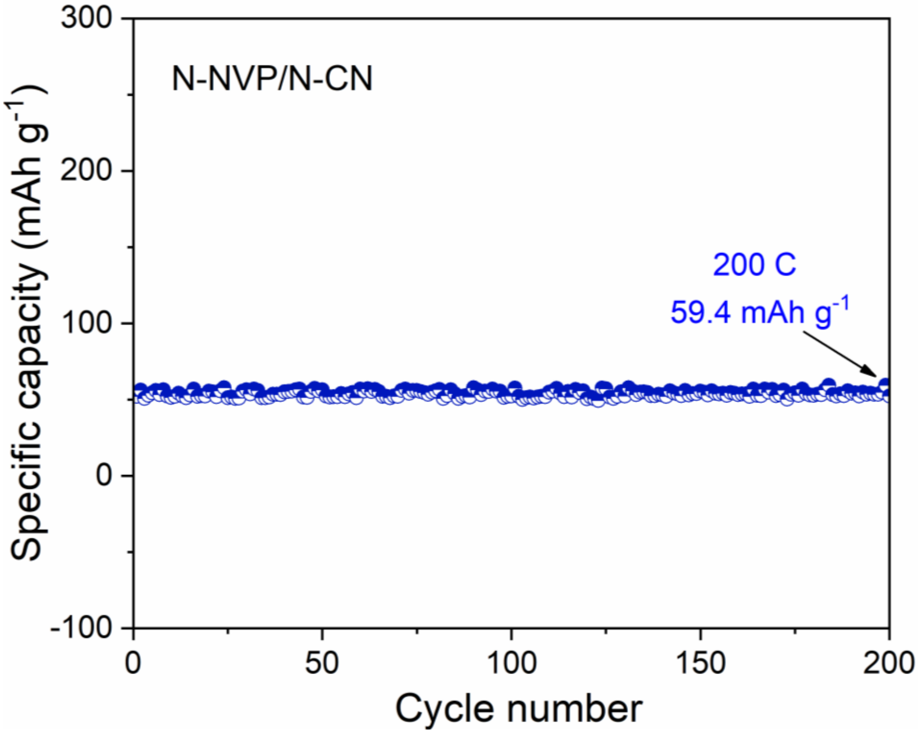

Figure S23. The long cycling stability of N-NVP/N-CN at high current densities of 200C.

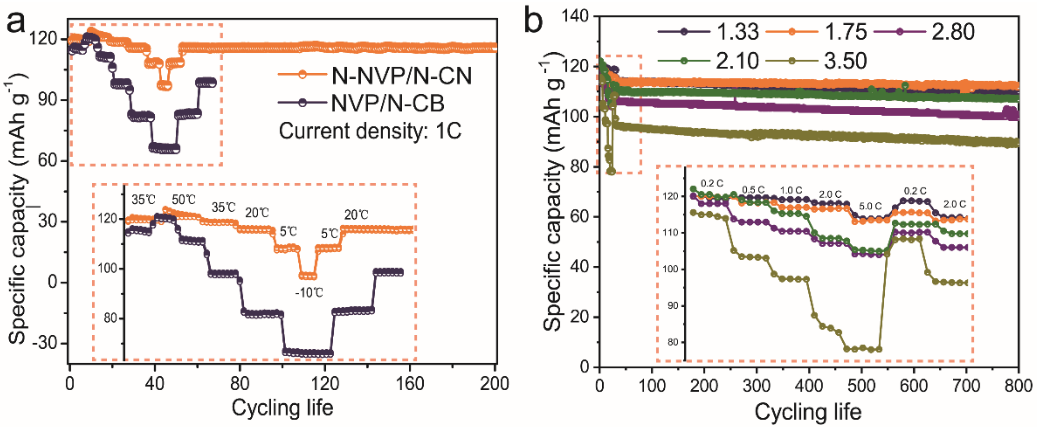

Figure S24. The cycling tests at different environment temperatures of N-NVP/N-CN and NVP-N-CB (a), and different loading masses (b) of N-NVP/N-CN.

As shown in Fig. S24a, compared with NVP/N-CB, the N-NVP/N-CN has presented a more stable capacity at 1 C at varying temperatures (from -10 °C and 50 °C), and a high capacity of 97.5 mAh g^{-1} was retained at a low temperature of -10 °C. When the temperature was switched back to 20 °C, a high capacity of 115.3 mAh g^{-1} was recovered, and there was almost no capacity fading after 200 cycles. The cycling stabilities at different loading masses were further measured. As shown in Fig. S24b, when the loading mass was set 1.75 mg cm^{-2}, there is no capacity fading at different increases of loading mass at different current densities. However, when the loading mass increased to more, the specific capacity faded sharply with the increase of loading mass at different current densities. When the loading mass was 1.75 mg cm^{-1} not only could satisfy the requirement of high energy density, but also higher specific capacity. It was mentioned that all the electrodes with different loading masses of N-NVP/N-CN achieved amazing cycling stability at 2 C.

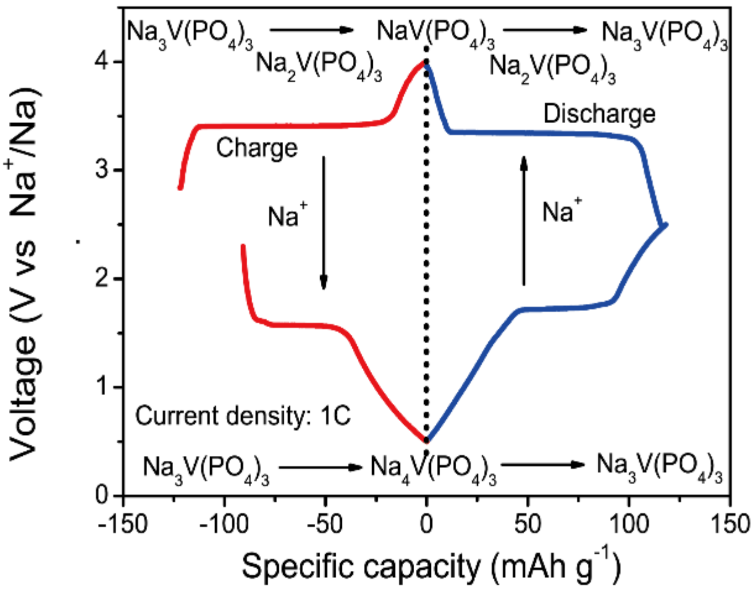

Figure S25. The charging/discharging curves at 1 C with a voltage window of 0.5-2.5 V and 2.5-3.8 V of N-NVP/N-CN.

As shown in Fig. S25, the N-NVP/N-CN exhibited a long charging or discharging platform with a discharge specific capacity of 90 mAh g^{-1} at a voltage window between

0.5 V and 2.5 V at 0.12 A g^{-1} (similar to 1 C). Thus, the P/N ratio of the N-NVP/N-CN//N-NVP/N-CN symmetric device was set as 3/4, and the capacity was calculated based on the mass of cathode.

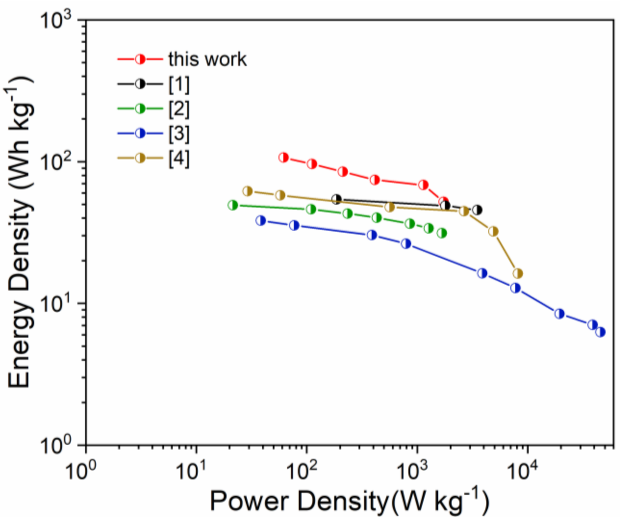

Figure S26. The comparison of Ragone plot with the previous symmetric cell reports.

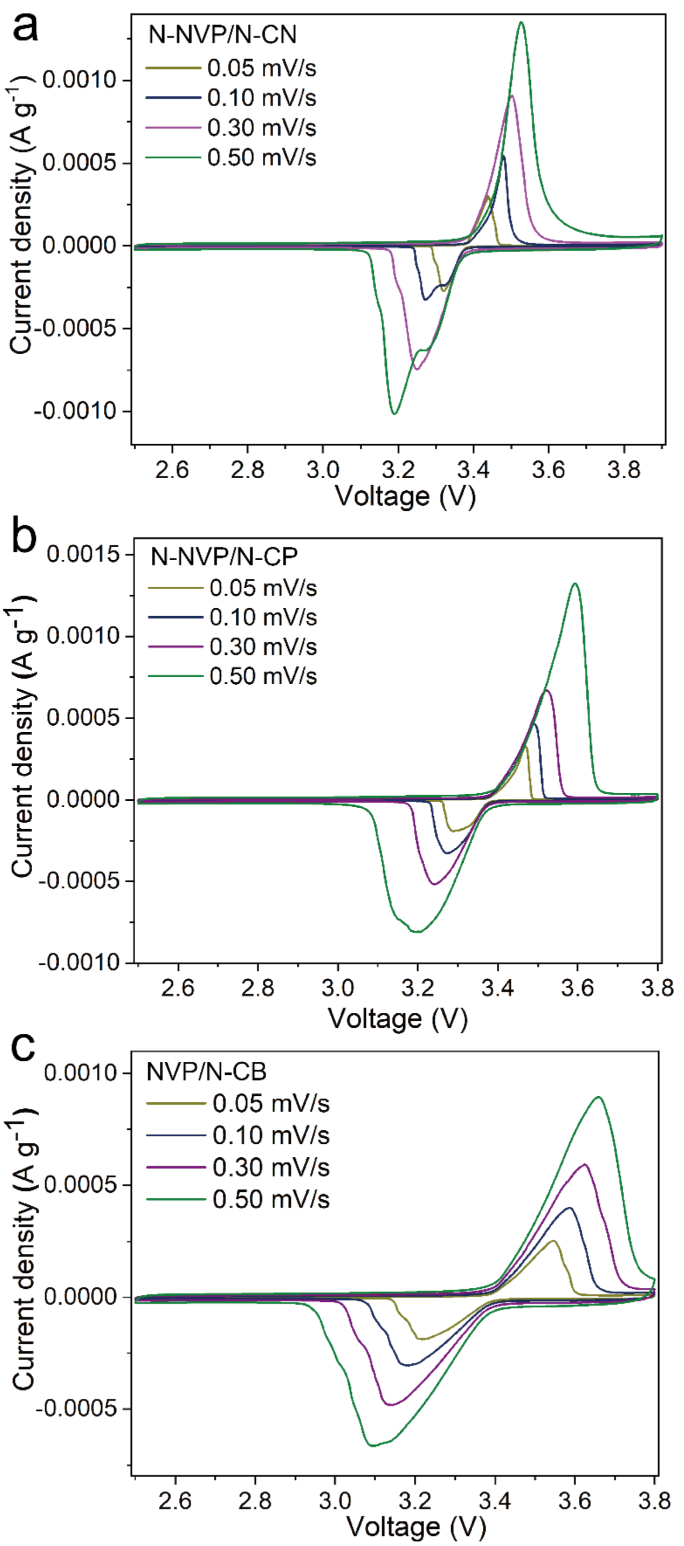

Figure S27. The CV curves at scan rates from 0.05 to 0.5 mV s^{-1} of N-NVP/N-CN (a), N-NVP/N-CP (b), and NVP/N-CB (c).

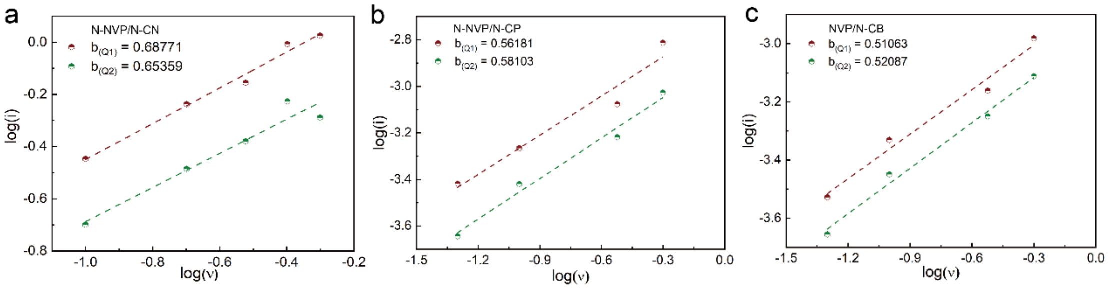

Figure S28. The linear relationship between normalized capacities (Q) and $v^{-1/2}$ according to cathode peaks in CV curves of N-NVP/N-CN (a), N-NVP/N-CP (b), and NVP/N-CB (c).

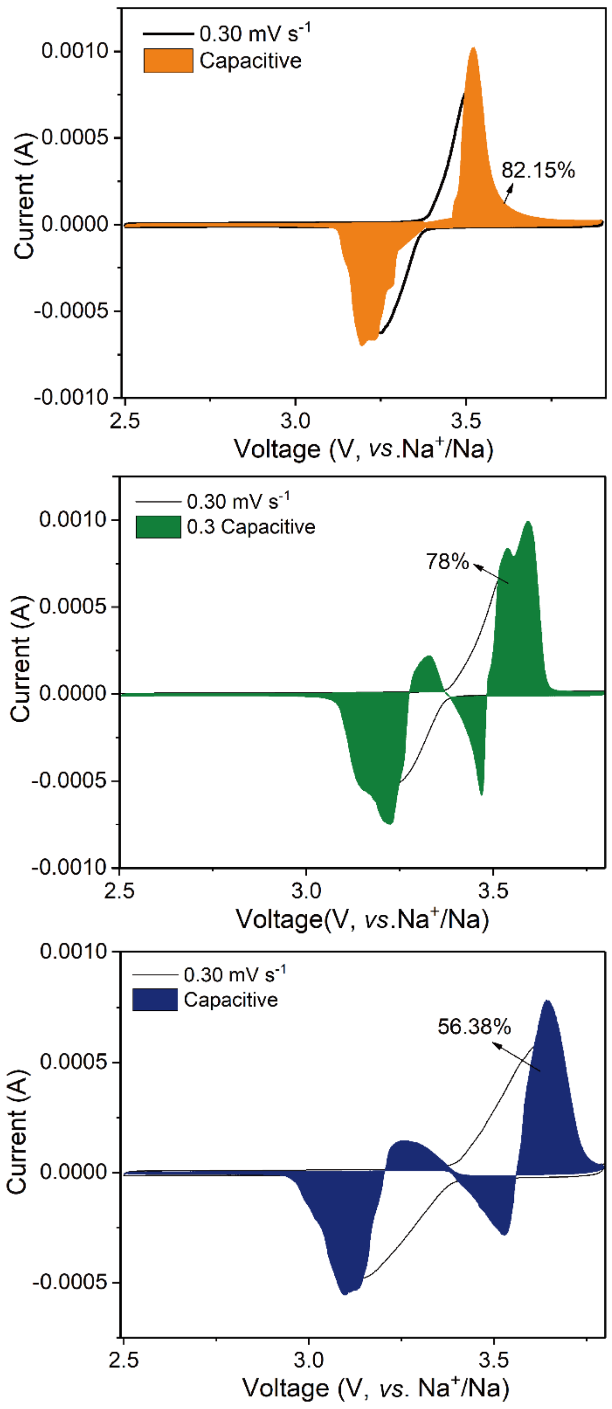

Figure S29. The capacitive contributions correspond to DCP and SCP of N-NVP/N-CN (a), N-NVP/N-CP (b), and NVP/N-CB (c).

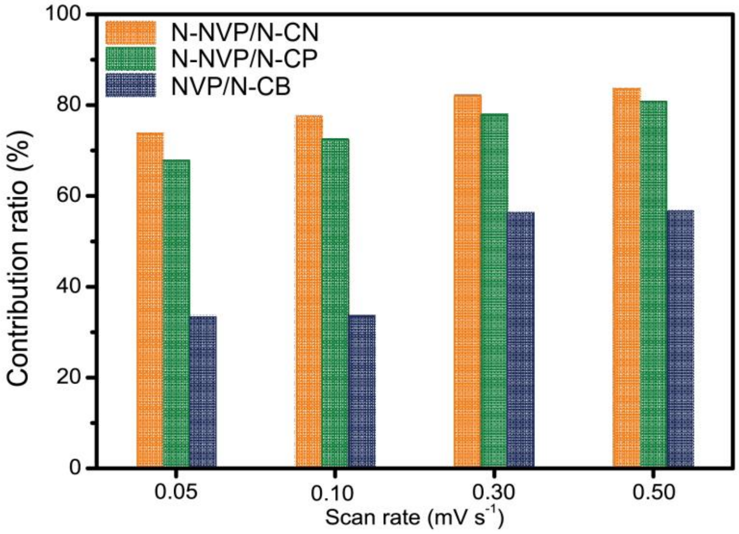

Figure S30. The capacitive contribution of N-NVP/N-CN, N-NVP/N-CP, and NVP/N-CB.

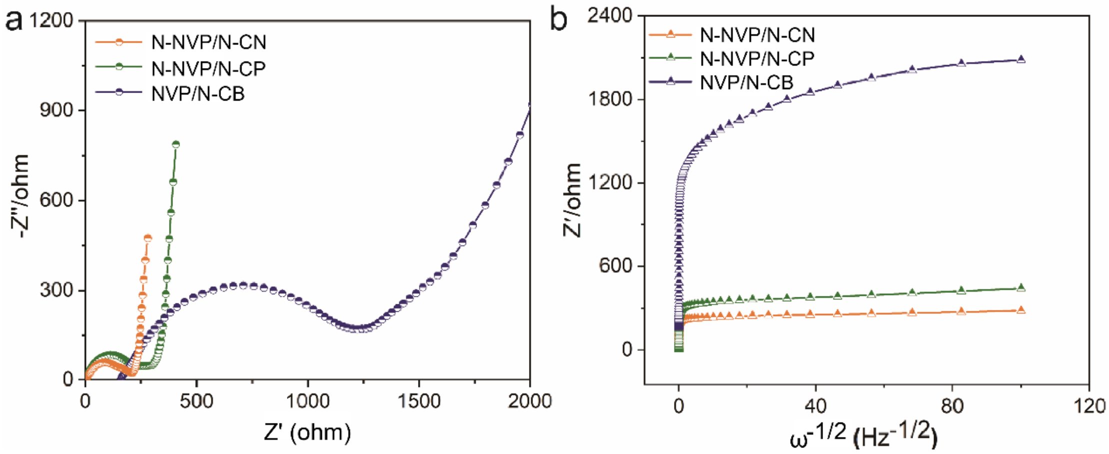

Figure S31. The electrochemical impedance (a) and the relation between $\omega^{-1/2}$ and Z' (i) (b) of N-NVP/N-CN, N-NVP/N-CP, and NVP/N-CB.

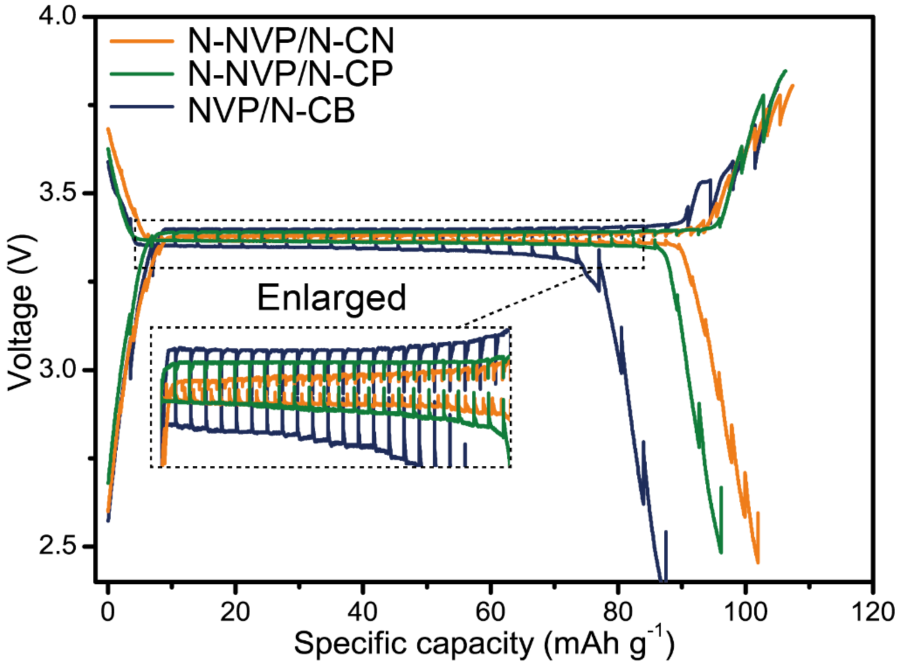

Figure S32. The GITT curves of N-NVP/N-CN, N-NVP/N-CP, and NVP/N-CB.

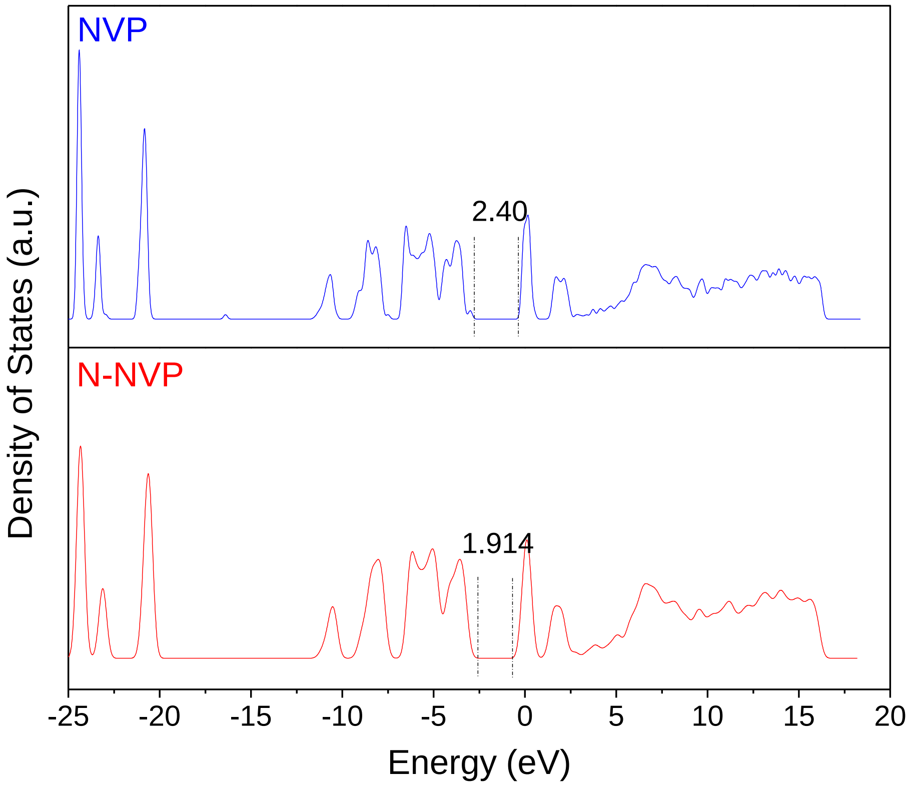

Figure S33. The DOS calculation of NVP and N-NVP.

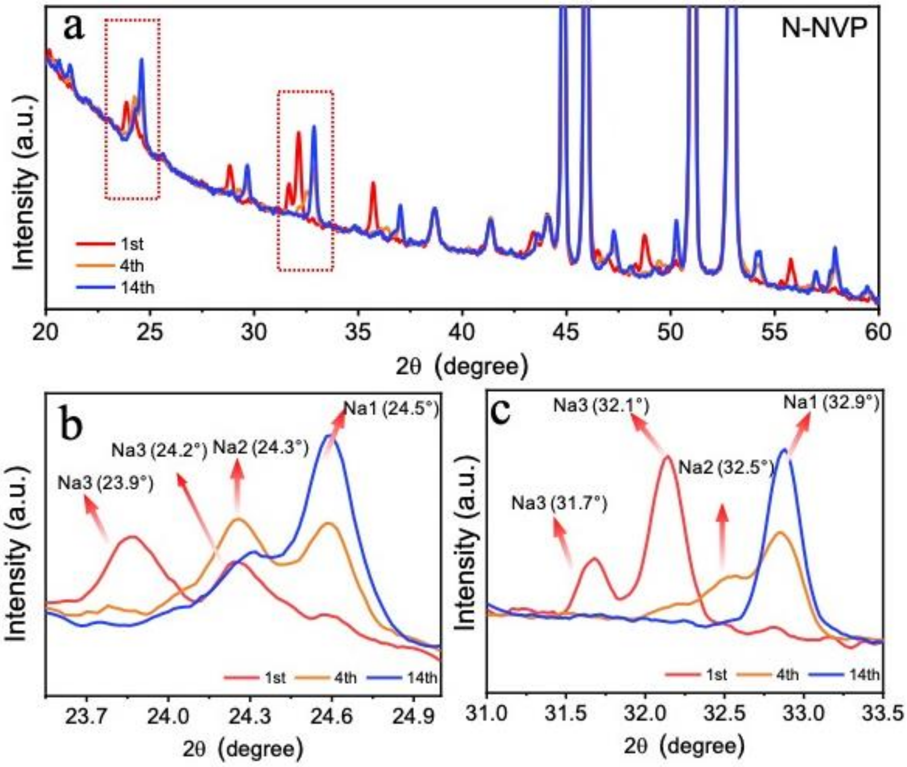

Figure S34. The typical *in-situ* XRD patterns of $Na_3V_2(PO_4)_3$ in the 1st scan, $Na_2V_2(PO_4)_3$ in the 4th scan and $NaV_2(PO_4)_3$ in the 14th, in which the three samples are marked as Na3, Na2, and Na1, respectively.

The *in-situ* XRD measurement was performed on a special cell using a pure beryllium foil as the X-ray scan window and a thin Al foil as the current collector with a Na block as anode *via* a reflecting mode.

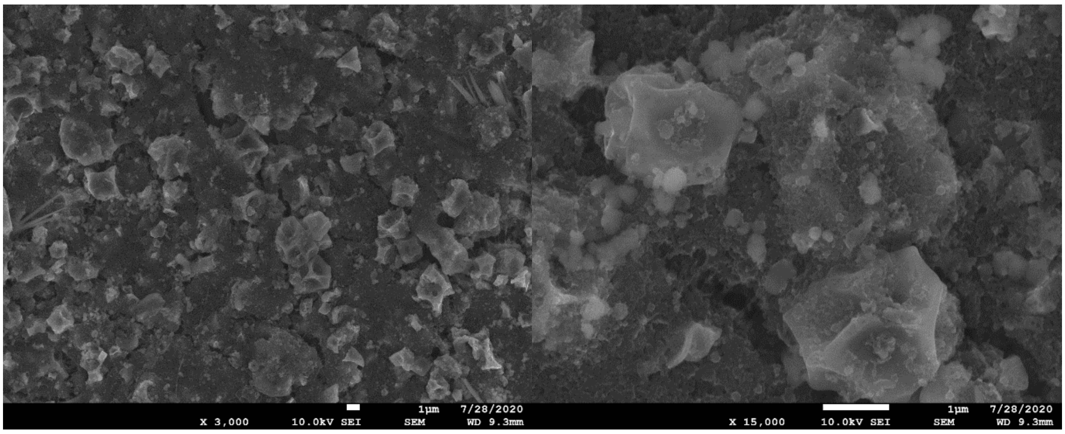

Figure S35. The SEM images of N-NVP/N-CP electrode after 2000 cycles.

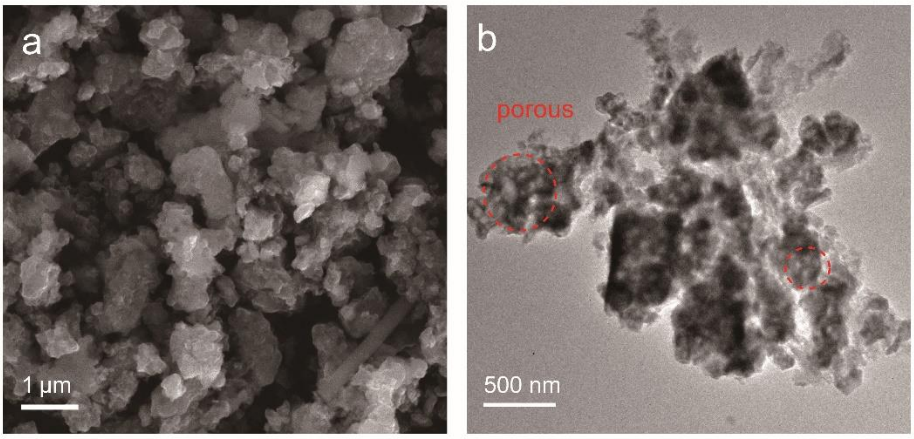

Figure S36. The SEM and TEM images of N-NVP/N-CN electrode after 2000 cycles.

We characterized the morphology of N-NVP/N-CN after long circulation. As shown in Fig. S36, the nanocage structure can be retained after a long circulation, the "breathable property" of nanocage structure is facilitating to realize the excellent cycling stability of the NVP electrode.

Table S1. The content of pyridine, pyrrole, and graphite type of nitrogen

	Pyridine N	Pyrrole N	Graphite N	Ratio
N-NVP/N-CN	1509	9000	468.32	3.22:19.22:1
N-NVP/N-CP	2640	5935	1228	2.45:4.83:1
NVP/N-CB	2311	7384	3108	1:3.2:1.34

Table S2. The NVP/N-CB cell parameter according to XRD refinement

Type	Fractional coordinates			Mult.	Occupancy
O1	0.141497	0.500980	0.080966	36	1.0000
O2	0.542792	0.844919	-0.025809	36	1.0000
Na1	0.333300	0.666700	0.166700	6	0.8050
Na2	0.666700	0.964669	0.083300	18	0.7310
P	-0.045419	0.333300	0.083300	18	1.0000

V	0.333300	0.666700	0.020116	12	1.0000
Cell parameter					
a	b	c	α	β	γ
8.729005	8.729005	21.808395	90.0000	90.0000	120.0000
Error					
R_{wp}/%		R_p/%		Average Integral	
7.02		5.38		0.933	

Table S3. The N-NVP/N-CP cell parameter according to XRD refinement

Type	Fractional coordinates			Multi	Occupancy
O1	0.137453	0.499884	0.079494	36	1.0204
O2	0.547748	0.845357	-0.023665	36	1.0871
V1	0.333330	0.666670	0.019101	12	0.9916
Na1	0.333330	0.666670	0.166670	6	0.8519
Na2	0.666670	0.963149	0.083330	18	0.7596
P1	-0.045905	0.333330	0.083330	18	1.0425
Cell parameter					
a	b	c	α	β	γ
8.726041	8.726041	21.823093	90.0000	90.0000	120.0000
Error					
R_{wp}/%		R_p/%		Average Integral	
8.25		6.42		0.893	

Table S4. The N-NVP/N-CN cell parameter according to XRD refinement

Type	Fractional coordinates			Multi	Occupancy
O1	0.139564	0.497858	0.079384	36	0.8701

N1	0.139564	0.497858	0.079384	36	0.2003
O2	0.545975	0.845561	-0.025518	36	0.8322
N2	0.545975	0.845561	-0.025518	36	0.2164
Na1	0.333300	0.666700	0.166700	6	0.8584
Na2	0.666700	0.965988	0.083330	18	0.7590
P	-0.043485	0.333300	0.083300	18	0.9902
V	0.333300	0.666700	0.019104	12	0.9900
Cell parameter					
a	b	c	α	β	γ
8.726089	8.726089	21.838377	90.0000	90.0000	120.0000
Error					
R_{wp}/%		R_p/%		Average Integral	
6.59		5.08		0.950	

Table S5. The relative ratio of N atom to O atom in N-NVP/N-CN, N-NVP/N-CP, and NVP/N-CB according to the results of XPS.

	N-NVP/C-B	N-NVP/C-HS	N-NVP/C-PHS
N1s	64766.90	11300.12	16197.36
O1s	1471263.49	83519.50	117884.57
Atomic ratio of N to O	2.00/28.37	2.02/9.624	2.92/13.61

Reference

[1] Y. Jiang, Y. Wu, Y. Chen, Z. Qi, J. Shi, L. Gu, Y. Yu, *Small* **2018**, *14*, 1703471.

[2] X. W. Liu, X. Y. Jiang, F. P. Zhong, X. M. Feng, W. H. Chen, X. P. Ai, H. X. Yang, Y. L. Cao, *ACS Appl. Mater. Interfaces* **2019**, *11*, 27833.

[3] M. K. Sadan, A. K. Haridas, H. H. Kim, C. Kim, G. B. Cho, K. K. Cho, J. H. Ahn, H. J. Ahn, *Nanoscale Adv.* **2020**, *2*, 5166.
[4] M. K. Sadan, M, Jeon, J. M. Yun, E. Song, K. K. Cho, J. H. Ahn, H. J. Ahn, *Sustain. Energ. Fuels* **2022**, *6*, 2155.
